# The hypervirulent Type-1/Type-17 phenotype of *Cryptococcus neoformans* clinical isolates is specific to A/J mice

**DOI:** 10.1128/iai.00585-24

**Published:** 2025-03-03

**Authors:** Minna Ding, Katrina M. Jackson, Madeline Harris-Gordon, Thamotharampillai Dileepan, David B. Meya, Kirsten Nielsen

**Affiliations:** 1 Department of Microbiology and Immunology, University of Minnesota 205104 , Minneapolis, Minnesota, USA; 2 Pathogen and Microbiome Institute, Northern Arizona University 3356 , Flagstaff, Arizona, USA; 3 Infectious Diseases Institute, College of Health Sciences, Makerere University 242941 , Kampala, Central Region, Uganda; 4 Department of Biomedical Sciences and Pathology, Virginia Tech 1757 , Blacksburg, Virginia, USA; University of California Davis, Davis, California, USA

**Keywords:** *Cryptococcus neoformans*, host-pathogen interactions, hypervirulence, damage-response framework, immune reconstitution inflammatory syndrome, Type 1 immune response, Type 17 immune response, cryptococcosis

## Abstract

*Cryptococcus neoformans* is a fungal pathogen that causes cryptococcal meningitis in immunocompromised individuals. Both host- and pathogen-specific factors are known to affect patient outcome, and recent studies showed that strain-specific differences in *C. neoformans* clinical isolates can influence virulence in A/J mice. However, it is unclear how the immunologic and genetic background of inbred mouse strains affects disease outcome during *C. neoformans* infection. In this study, we show that a hypervirulent phenotype is dependent on the host immune response and mouse genetic background. A/J mice intranasally infected with the hypervirulent isolates, UgCl247, UgCl422, and UgCl236, have increased neutrophil and T-cell recruitment when compared with infection with the reference strain KN99α. In addition, the cytokine profile of the hypervirulent isolates in A/J mice had a profound IFNγ and IL-17 response, and lung resident CD4 T-cells isolated from A/J mice expressed significantly increased Th1 (CXCR3, Tbet) and Th17 (RORγT) markers compared with KN99α infection. Intriguingly, when C57BL/6J mice were infected with these isolates, the hypervirulent phenotype was not evident, and all isolates had virulence comparable to the KN99α control. The immune response in C57BL/6J mice was also nearly identical in response to infections with the hypervirulent isolates and the KN99α control strain. Finally, we determined that the hypervirulent phenotype in A/J mice is not caused by known genetic mutations in the A/J inbred mouse background. Overall, this study demonstrates that an inbred mouse inhalation model can be used to identify host- and pathogen-specific factors that affect *C. neoformans* disease progression.

## INTRODUCTION

*Cryptococcus neoformans* is an opportunistic fungal pathogen that causes life-threatening cryptococcal meningitis in immunocompromised individuals. Despite improvements in standard of care, a 2020 estimate of the global burden of HIV-associated cryptococcal infection found that cryptococcal disease still accounts for 19% of all AIDS-related deaths (1). As such, there is still an urgent need to develop more effective antifungal therapies against cryptococcal meningitis. Given that cryptococcal meningitis has highly variable disease presentation and patient outcome, new antifungal therapeutics will benefit from a precision-based medical approach that integrates both patient- and pathogen-specific factors.

A two-pronged approach aimed at understanding the interplay between host- and pathogen-specific factors is already underway within the larger *Cryptococcus* research community. There is a plethora of evidence showing association between patient immune response and variability in disease manifestation (2–7). At the same time, other studies have found that pathogen-specific factors also play a significant role in causing variable disease presentation (8–14). A previous study demonstrated that normalizing the host background using the mouse inhalation model with A/J mice made it possible to recapitulate patient outcome using clinical isolates and identify pathogen-specific factors that influence severity of disease progression (15). However, a separate study observed an inverse relationship between human and A/J mouse outcome, suggesting that the association between human and mouse survival with clinical isolates depends on the strain genotype (16). There is still much to unravel concerning the mechanism by which different *C. neoformans* strain genotypes affect virulence and the host immune response.

A recent study explored the association between strain genotype, virulence, and immune response using a library of clinical isolates and observed a spectrum of virulence in A/J mice ranging from latency to hypervirulence (17). Of note, the study defined hypervirulent clinical isolates as isolates that had a median survival in A/J mice occurring 2 days prior to median survival in the KN99α reference strain (17). These clinical isolates, UgCl247, UgCl236, and UgCl422, displayed two notable phenotypes in their respective patient cohorts and in A/J mice: all isolates were collected from patients who survived after developing cryptococcal meningitis and had profoundly increased IFNγ levels compared with KN99α infection in the lungs of A/J mice (17). IFNγ has historically been considered protective in most cases of human *C. neoformans* infection (18–20). The association between the hypervirulent phenotype in A/J mice and elevated IFNγ cytokine signature was unexpected and suggested that the host-specific factors that contribute to the disease progression of *C. neoformans* clinical isolates are much more complex than previously established.

In the current study, we sought to investigate the cause of the hypervirulent phenotype in A/J mice through comparison of immune responses and hypervirulence phenotypes across various inbred mouse strains. We found that mouse genetic background influences the immune response and survival of the hypervirulent isolates. Our data indicate that the inbred mouse inhalation model can be used to interrogate host- and pathogen-specific factors that affect *C. neoformans* disease progression.

## RESULTS

### Hypervirulent isolates do not disseminate faster than KN99α

**Fig 1:**
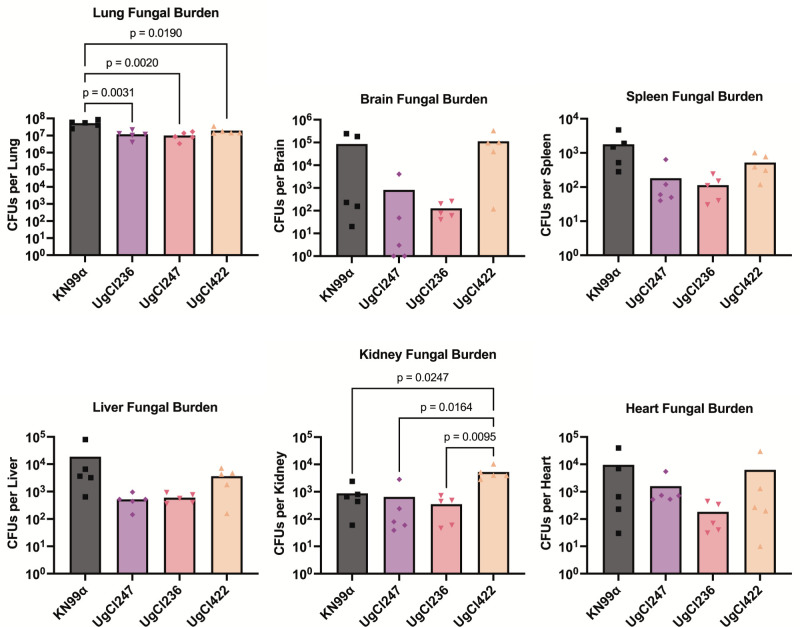
Hypervirulent isolates do not disseminate faster than KN99α in A/J mice. A/J mice were intranasally infected with *C. neoformans* KN99α (*n* = 5 mice), UgCl247 (*n* = 5 mice), UgCl236 (*n* = 5 mice), or UgCl422 (*n* = 5 mice). At 17 days post-infection, mice were sacrificed, and lung, brain, spleen, liver, kidneys, and hearts were removed. Each organ was plated for colony-forming units (CFUs). Significance was determined using one-way ANOVA with Bonferroni correction.

We previously identified three *C. neoformans* clinical isolates that were hypervirulent in A/J mice: UgCl247, UgCl422, and UgCl236 (17). These isolates were defined as hypervirulent due to a median mortality of at least 2 days prior to that of the KN99α reference strain in A/J mice (17). To further characterize the hypervirulent phenotype, we tested the hypothesis that hypervirulence was due to the development of a more rapid systemic infection. We analyzed fungal dissemination to multiple organs in A/J mice infected with each of the three hypervirulent isolates. We observed significantly decreased lung fungal burden compared with KN99α infection, but no other consistent difference in brain, spleen, liver, kidney, and heart fungal burden among the hypervirulent isolates (Fig. 1). These findings led us to conclude that the hypervirulent phenotype was not due to more rapid organ dissemination. Instead, the significantly decreased lung fungal burden in the hypervirulent infections indicated that the pulmonary host response was a major contributor toward rapid mortality.

### The hypervirulence phenotype was specific to the A/J inbred genetic background

**Fig 2:**
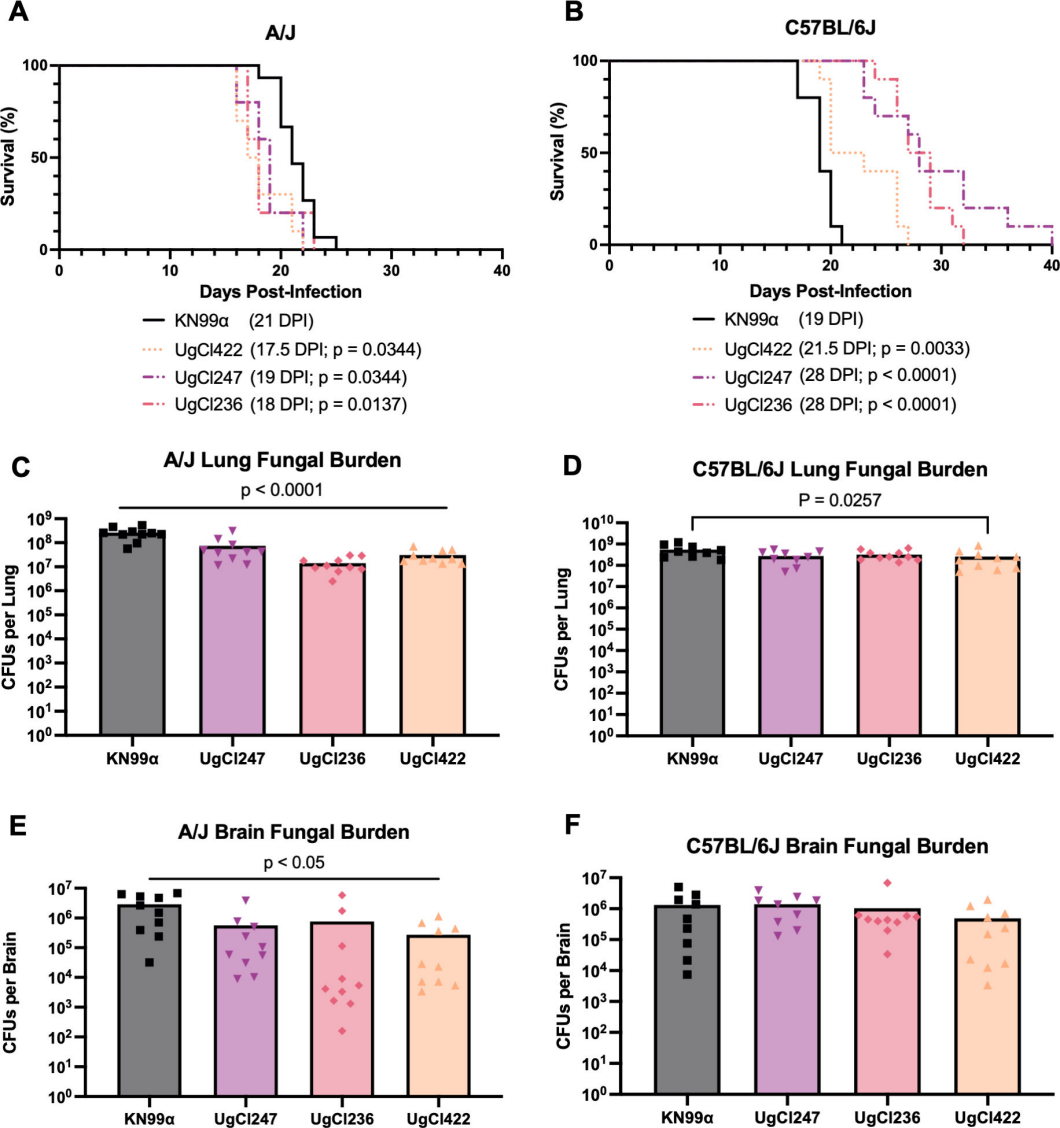
UgCl247, UgCl422, and UgCl236 were not hypervirulent when infected in C57BL/6J mice. (**A**) A/J (*n* = 10 mice per strain) and (**B**) C57BL/6 (*n* = 10 mice per strain) mice were intranasally infected with *C. neoformans* UgCl247, UgCl236, UgCl422, and KN99ɑ. Mice were monitored for signs of morbidity and euthanized at terminal endpoint (20% total weight loss, 1 g/day weight loss for two consecutive days; or neurological symptoms including loss of sternal recumbency, partial paralysis, seizure, convulsion, or coma). At terminal endpoint, (**C**) the lungs and (**D**) brain of A/J mice were harvested, homogenized, and plated for colony-forming units (CFUs). CFUs were also collected from the (**E**) lungs and (**F**) brain of C57BL/6J mice. Kaplan–Meier survival kinetics were analyzed using log-rank testing. Median survival is denoted in parenthesis by days post-infection (DPI) with significance values from comparison to KN99ɑ infection. *P*-values by one-way ANOVA with Bonferroni correction.

To determine if the host genetic background affected the virulence of the hypervirulent isolates, we infected both A/J mice and C57BL/6J mice with UgCl247, UgCl422, UgCl236, or KN99α and assessed for survival and terminal endpoint fungal burden. The median survival for all hypervirulent isolates was significantly different compared with KN99α in A/J mice, and all isolates had a median survival of at least 2 days prior to that of KN99α (Fig. 2A). In contrast, the median survival of the hypervirulent isolates in C57BL/6J mice was significantly prolonged compared with KN99α infection (Fig. 2B). Thus, the hypervirulence phenotype observed in A/J mice was lost when C57BL/6J mice were infected with the isolates.

Upon reaching terminal endpoint criteria, the lung fungal burden of the hypervirulent isolates was significantly decreased compared with KN99α in A/J mice (Fig. 2C). Conversely, the terminal lung fungal burden of only UgCl422 was significantly decreased compared with KN99α in C57BL/6J mice (Fig. 2D). The terminal brain fungal burden of all hypervirulent isolates was also significantly decreased compared with KN99α in A/J mice (Fig. 2E). However, no difference in terminal brain fungal burden was observed between the hypervirulent isolates and KN99α in C57BL/6J mice (Fig. 2F). Given that all hypervirulent isolates had significantly decreased lung and brain fungal burden compared with KN99α, it was likely that the host factor(s) contributing to hypervirulence in A/J mice was related to their inbred genetic background.

**Fig 3:**
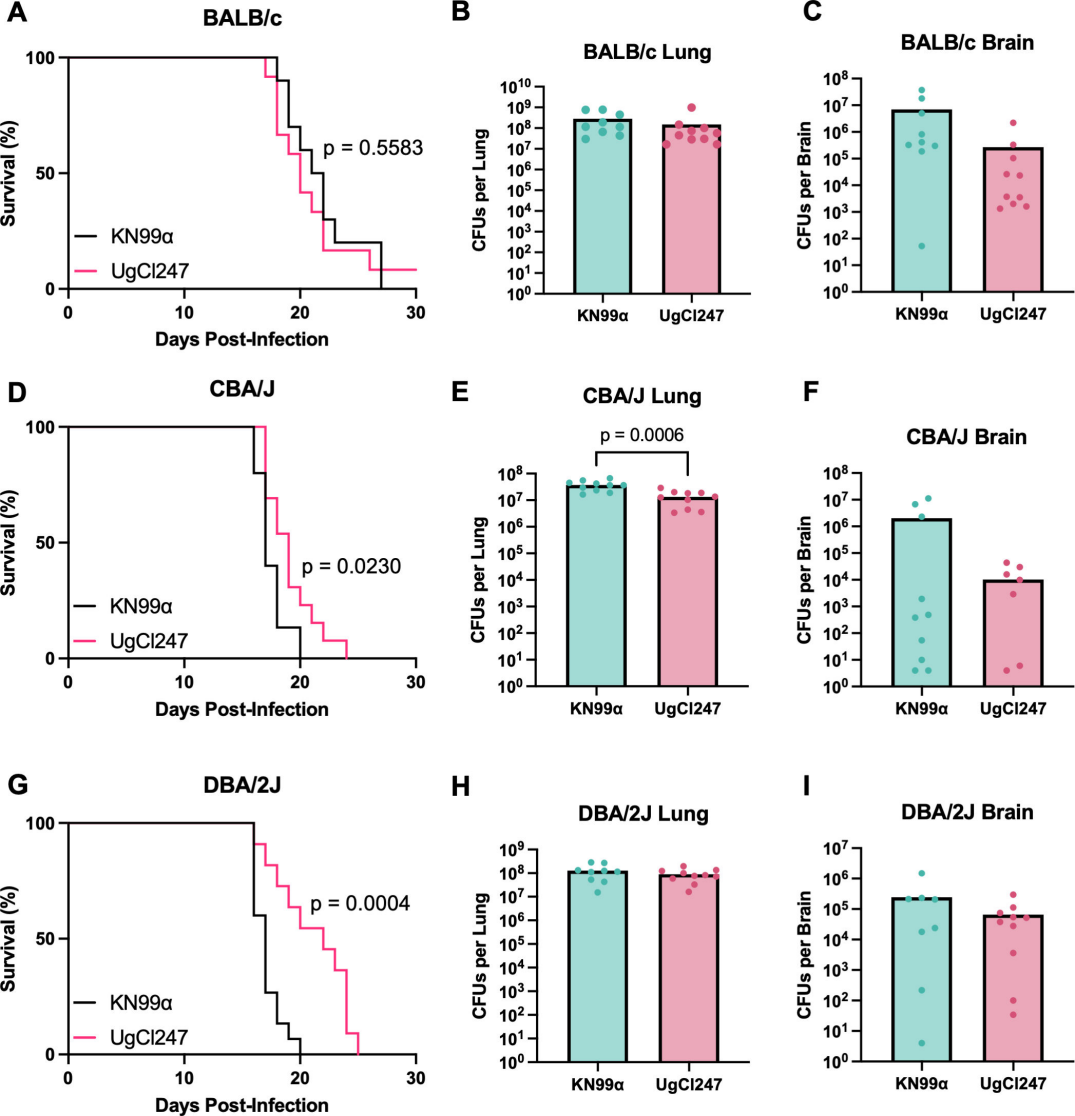
UgCl247 is not hypervirulent when infected in BALB/c, CBA/J, and DBA/2J mice. (**A**) BALB/c mice (*n* = 9–10 mice per strain) were intranasally infected with *C. neoformans* UgCl247 and KN99ɑ. Mice were monitored for signs of morbidity and euthanized at terminal endpoint (20% total weight loss, 1 g/day weight loss for two consecutive days; or neurological symptoms including loss of sternal recumbency, partial paralysis, seizure, convulsion, or coma). At terminal endpoint, (**B**) the lungs and (**C**) brain of BALB/c mice were harvested, homogenized, and plated for colony forming units (CFUs). The same experimental setup was performed for (**D–F**) CBA/J mice (*n* = 10 mice per strain) and (**G–I**) DBA/2J mice (*n* = 9–10 mice per strain), respectively. Kaplan–Meier survival kinetics were analyzed using log-rank testing. *P*-value calculated by two-tailed *t*-test.

**Fig 4:**
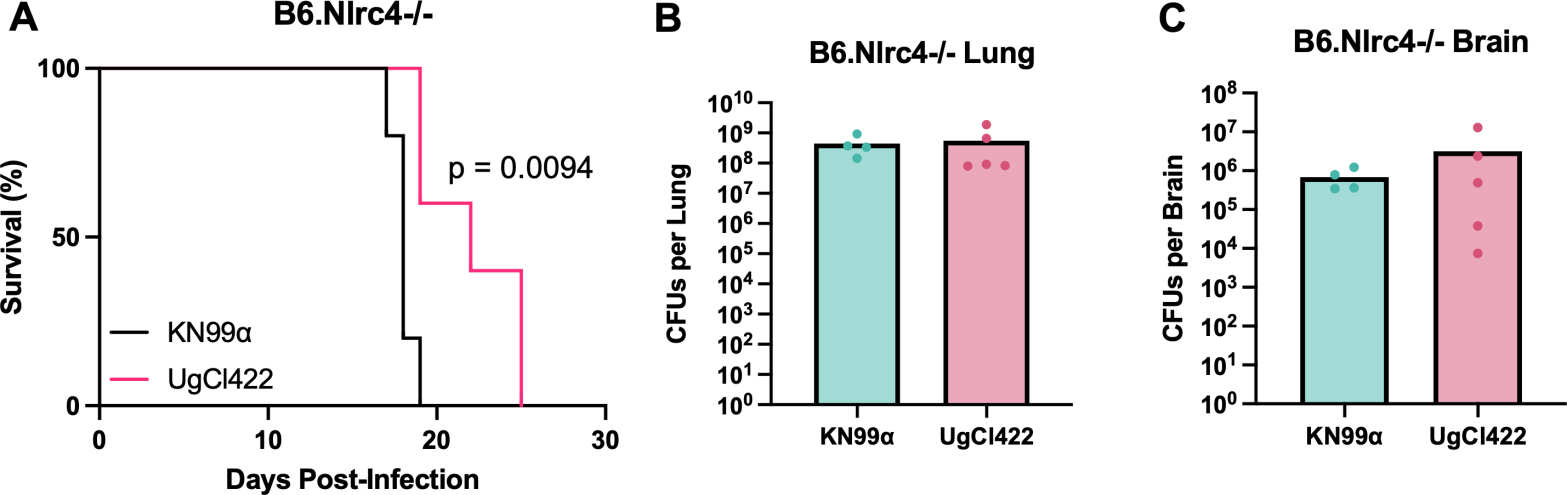
UgCl422 is not hypervirulent when infected in NLRC4-deficient mice. B6.Nlrc4-/- mice (*n* = 5 mice per strain) were intranasally infected with *C. neoformans* UgCl422 and KN99ɑ. Mice were monitored for signs of morbidity and euthanized at terminal endpoint (20% total weight loss, 1 g/day weight loss for two consecutive days; or neurological symptoms including loss of sternal recumbency, partial paralysis, seizure, convulsion, or coma). (**A**) Kaplan–Meier survival kinetics was analyzed using log-rank testing. At the terminal endpoint, (**B**) the lungs and (**C**) brain of B6.Nlrc4-/- mice were harvested, homogenized, and plated for colony-forming units (CFUs). One out of five B6.Nlrc4-/- mice succumbed to KN99ɑ infection overnight, and its organs could not be processed for fungal burden. *P*-value calculated by two-tailed *t*-test.

As shown in Table 1, the A/J inbred mouse strain has three known genetic mutations that may influence the immune response to *C. neoformans*: (i) a *Hc^0^* loss-of-function mutation in complement component 5 (C5), (ii) a *Nlrp1b^R/R^* mutation that confers resistance to anthrax lethal toxin, and (iii) a defective *Naip5* allele involved in NLRC4 inflammasome activation (21). To determine if these known genetic mutations were contributing to the hypervirulence phenotype, we infected inbred BALB/c, CBA/J, and DBA/2J mice that have different alleles of the three aforementioned genetic loci with a representative hypervirulent isolate, UgCl247, or the control strain KN99α, and assessed survival and terminal fungal burden (Table 1). We found that there was no difference in median survival, terminal lung fungal burden, and terminal brain fungal burden for BALB/c (C5 sufficient and *Nlrp1b^s/s^*) mice infected with UgCl247 compared with KN99α (Fig. 3A through C). Both CBA/J (C5 sufficient and *Nlrp1b^s/s^*) and DBA/2J (C5 deficient and *Nlrp1b^R/R^*) mice infected with UgCl247 had significantly higher median survival compared with KN99α (Fig. 3D and 4G). While the terminal lung fungal burden was significantly decreased in UgCl247 infection, the terminal brain fungal burden was nonsignificant between UgCl247 and KN99α in CBA/J mice (Fig. 3E and F). Like with BALB/c mice, no difference between UgCl247 and KN99α infection in DBA/2J mice was observed for terminal lung and brain fungal burden (Fig. 3H and I). Thus, our data showed that complement C5 deficiency and *Nlrp1b^R/R^* mutation (Table 1) did not promote the hypervirulent phenotype observed during UgCl247 infection in A/J mice.

**TABLE 1:** Survival and fungal burden outcomes of UgCl247 infection based on known genetic mutations in A/J, C57BL/6J, BALB/c, CBA/J, and DBA/J mice

Strains	Complement C5	NOD-like receptor proteins	Neuronal apoptosis inhibitory proteins (NAIPs)	Median survival of UgCl247 compared with KN99α	Terminal fungal burden of UgCl247 compared with KN99α
**A/J**	*Hc^0^*	*Nlrp1b^R/R^*	Defective *Naip5* allele	2 days prior to KN99α^*a*^	Lung and brain fungal burden significantly decreased
**C57BL/6J**	Sufficient	*Nlrp1b^R/R^*	*Naip1, Naip2, Naip5*, and *Naip6*	9 days after KN99α^*a*^	No significant difference in lung and brain fungal burden.
**BALB/c**	Sufficient	*Nlrp1b^s/s^*	No known deficiencies/mutations; *Naip4*	1 day prior to KN99α^*b*^	No significant difference in lung and brain fungal burden
**CBA/J**	Sufficient	*Nlrp1b^s/s^*	No known deficiencies/mutations	2 days after KN99α^*a*^	Only lung fungal burden significantly decreased.
**DBA/2J**	*Hc^0^*	*Nlrp1b^R/R^*	No known deficiencies/mutations	5 days after KN99α^*a*^	No significant difference in lung and brain fungal burden

^*a*^ Indicates a significant difference between UgCl247 and KN99α infections.

^*b*^ Indicates no significant difference between UgCl247 and KN99α infections.

NAIP5 is known to activate the NLRC4 inflammasome pathway via recognition of bacterial flagellin (22). To determine if the functional defects in the *Naip5* gene found in the inbred A/J mouse strain contributed to hypervirulence, we infected B6.Nlrc4-/- mice with either a representative hypervirulent isolate, UgCl422, or the control strain KN99α and assessed the infections for survival and terminal fungal burden. We found that B6.Nlrc4-/- mice infected with UgCl422 had significantly increased median survival compared with KN99α infection (Fig. 4A). In addition, there was no significant difference in terminal lung and brain fungal burden (Fig. 4B and C). Thus, NAIP5 deficiency via NLRC4 inflammasome activation did not contribute to the hypervirulent phenotype observed in A/J mice.

While we were unable to determine the exact host genetic factor(s) contributing to hypervirulence, it is clear that this phenotype of IFNγ-associated hypervirulent isolates was specific to the A/J inbred genetic background.

### Hypervirulence in A/J mice can be attributed to the pulmonary immune response

**Fig 5:**
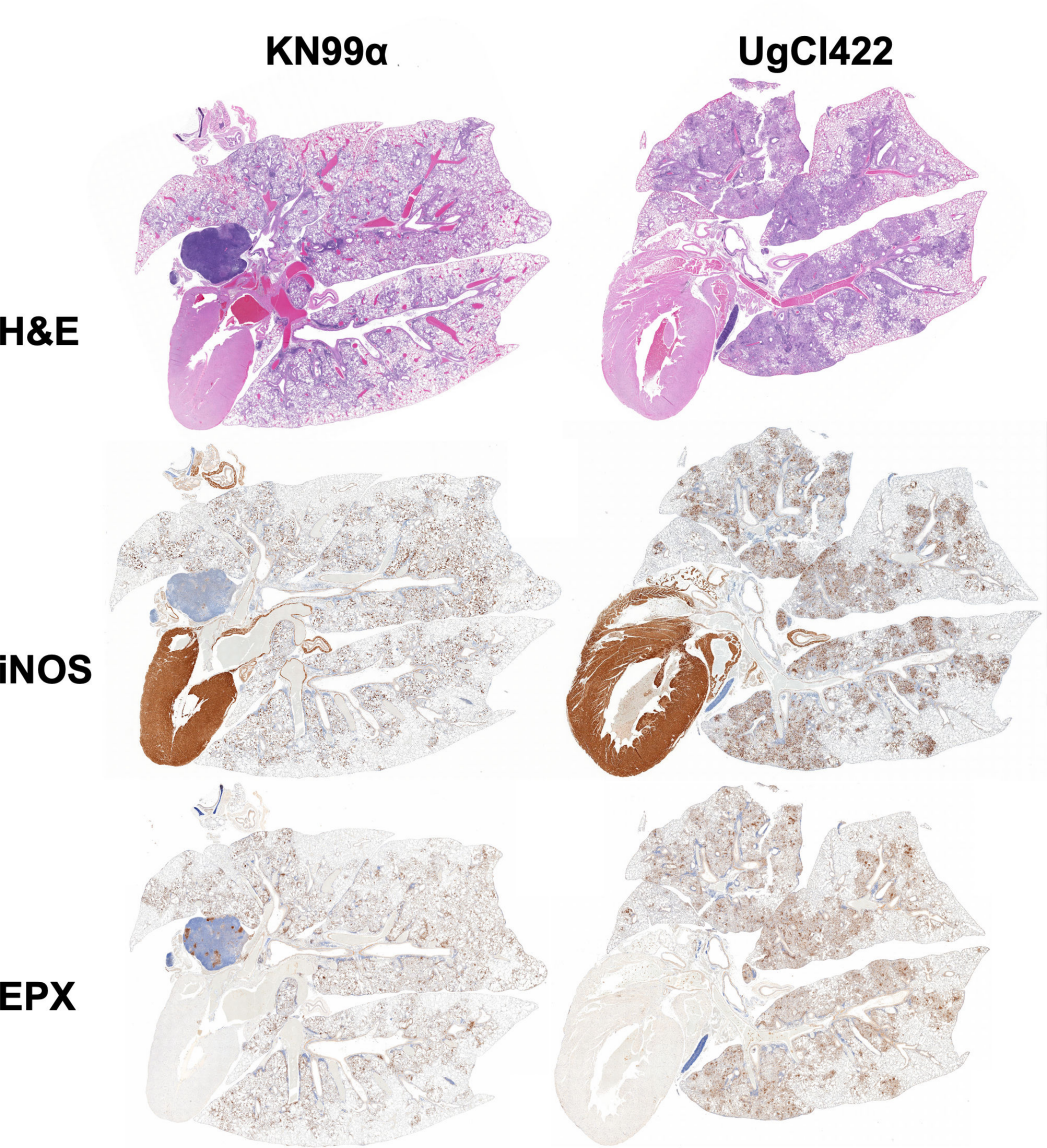
Histopathology of A/J mouse lungs infected with KN99α and UgCl422. A/J mice (*n* = 3 mice) were infected with *C. neoformans* KN99α or UgCl422. At 17 days post-infection, mice were sacrificed, and the lungs were placed in formalin and processed for histopathology. Lung sections were stained with hematoxylin and eosin stain (H&E), inducible nitric oxide synthase (iNOS), or eosinophil peroxidase (EPX). Representative lung images are shown.

Previously, cytokine analysis of the lung homogenate from A/J mice infected with UgCl247, UgCl422, and UgCl236 was associated with increased IFNγ levels (17). Given that the hypervirulent phenotype was specific to the A/J mouse genetic background, we wanted to better characterize the pulmonary inflammatory response against hypervirulent isolates in A/J mice. We infected A/J mice with a representative hypervirulent isolate, UgCl422, or the reference strain KN99α and analyzed the lung immune response via histopathology. Based on hematoxylin and eosin (H&E) staining, we observed increased inflammatory infiltration into the lung parenchyma in UgCl422 infection compared with KN99α infection (Fig. 5). Furthermore, lung tissue sections from A/J mice infected with UgCl422 had increased nitric oxide synthase (iNOS) and eosinophil peroxidase (EPX) staining compared with those of KN99α (Fig. 5). Since iNOS is commonly produced by classically activated macrophages and EPX is associated with eosinophil activity, these findings suggested that mice infected with UgCl422 had increased macrophage and eosinophil recruitment to the lungs. Altogether, we confirmed that the hypervirulent isolates elicit a different pulmonary inflammatory response compared with KN99α infection in A/J mice.

### Type-1/Type-17 immune response during hypervirulent infection is specific to A/J mice

**Fig 6:**
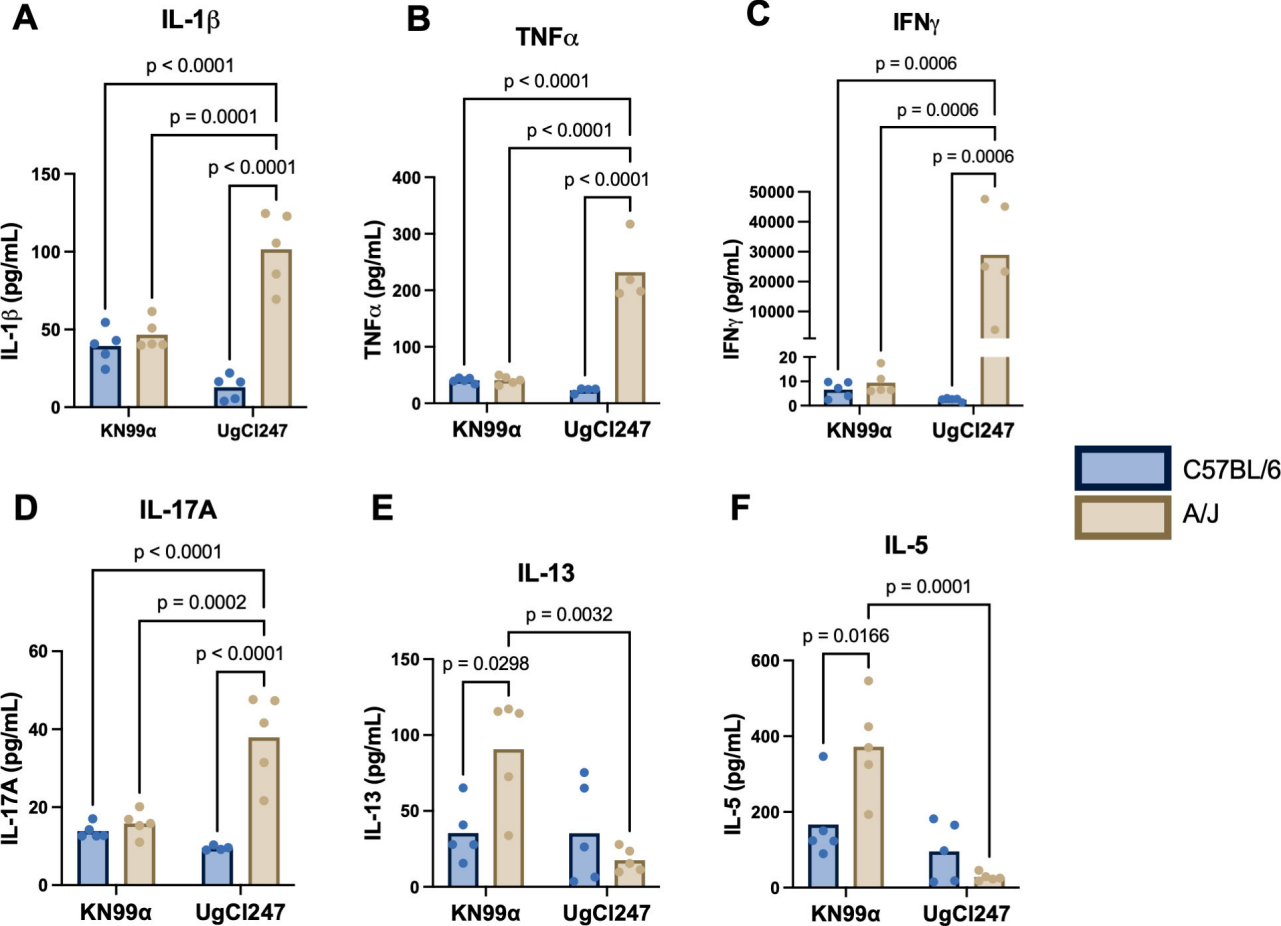
Differential cytokine response to UgCl247 in A/J and C57BL/6J mice. A/J (*n* = 5 mice per strain) and C57BL/6J (*n* = 5 mice per strain) mice were intranasally infected with *C. neoformans* UgCl247 or KN99ɑ. At 17 days post-infection, mice were euthanized, lungs were isolated, homogenized, and lung supernatant was collected. Cytokine levels for (**A**) IL-1β, (**B**) TNF-α, (**C**) IFNγ, (**D**) IL-17A, (**E**) IL-13, and (**F**) IL-5 were measured using a Th1/Th2/Th9/Th17/Th22/Treg Luminex panel. *P*-values calculated by one-way ANOVA with Bonferroni correction.

To identify the host immune factor(s) responsible for driving the hypervirulence phenotype, we infected both A/J and C57BL/6J mice with a representative hypervirulent isolate, UgCl247, or the control strain KN99α and assessed for cytokine production. We observed significantly increased production of the pro-inflammatory cytokines IL-1β and TNF-α in the lungs of A/J mice infected with UgCl247 compared with all other experimental conditions (Fig. 6A and B). In addition, we also observed significantly increased production of IFNγ and IL-17A in the lungs of A/J mice infected with UgCl247 compared with all other experimental conditions (Fig. 6C and D), indicating that the hypervirulent infection in A/J mice was driven by a Type-1/Type-17 immune response. Conversely, C57BL/6J mice infected with UgCl247 had significantly increased IL-13 and IL-5 production compared with C57BL/6J mice infected with KN99α and A/J mice infected with UgCl247 (Fig. 6E and F), indicating that C57BL/6J mice infected with UgCl247 have a type 2 skewed immune response. Furthermore, the cytokine response between UgCl247 and KN99α infection in BALB/c, CBA/J, and DBA/J mice did not exhibit any significant differences, aside from a significant increase in the production of IFNγ during UgCl247 infection compared with KN99α infection in CBA/J mice (Fig. S1). Based on these findings, we determined that the Type-1/Type-17 immune polarization observed in A/J mice infected with UgCl247 was specific to the hypervirulence phenotype.

**Fig 7:**
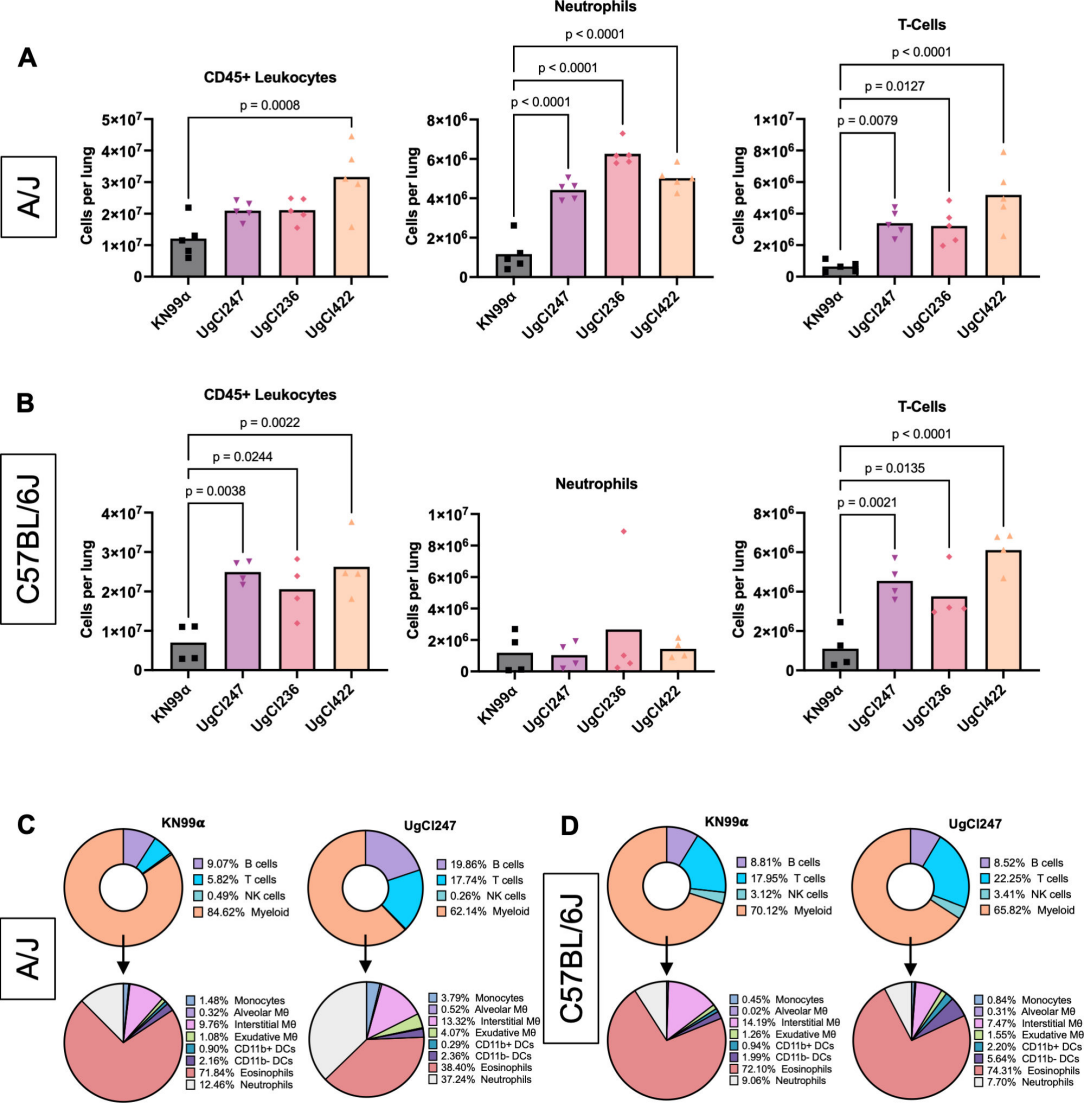
Pulmonary immune response to hypervirulent isolates in A/J mice. (**A**) A/J mice were intranasally infected with *C. neoformans* UgCl247 (*n* = 5 mice), UgCl422 (*n* = 5 mice), UgCl236 (*n* = 5 mice), and KN99ɑ (*n* = 5 mice). (**B**) C57BL/6J mice were intranasally infected with UgCl247 (*n* = 4 mice), UgCl422 (*n* = 4 mice), UgCl236 (*n* = 4 mice), and KN99ɑ (*n* = 4 mice). Single-cell lung suspensions were generated at 17 days post-infection. CD45+ leukocyte cell populations were quantified using flow cytometry. *P*-values calculated via one-way ANOVA with Bonferroni correction. (**C**) Representative mean immune cell proportions of KN99ɑ (*n* = 5 mice) and UgCl247 (*n* = 5) infection in A/J mice and (**D**) KN99ɑ (*n* = 4 mice) and UgCl247 (*n* = 4) infection in C57BL/6J mice were presented in pie charts. Proportion of B-cells, T-cells, NK cells, and myeloid cells were calculated by dividing the total number of lung CD45+ cells. Proportion of monocytes, alveolar macrophages (Mθ), interstitial Mθ, exudative Mθ, CD11b + DCs, CD11b- DCs, eosinophils, and neutrophils were calculated by dividing the total number of myeloid cells.

To further characterize the host immune response, we used flow cytometry to analyze the pulmonary immune cells generated in A/J and C57BL/6J mice during infection with UgCl247, UgCl236, and UgCl422. Consistent with our IL-17A cytokine data, all of the hypervirulent isolates elicited a significant increase in neutrophils in the lungs of infected A/J mice compared with KN99α (Fig. 7A). No difference in neutrophil response between KN99α and the hypervirulent infections was observed in C57BL/6J mice (Fig. 7B). Unlike in A/J mice, the total number of CD45+ leukocytes generated during infection with the hypervirulent isolates in C57BL/6J mice was significantly increased compared with KN99α infection (Fig. 7A and B). There was no consistent pattern of recruitment of monocytes, macrophages, dendritic cells (DCs), natural killer (NK) cells, eosinophils, or B-cells among the hypervirulent isolate infections in both A/J and C57BL/6J mice (Fig. S2 and S3). Overall, neutrophils were the dominant myeloid cell type during UgCl247 infection in A/J mice, while eosinophils were the dominant myeloid cell type during UgCl247 infection in C57BL/6J mice (Fig. 7C and D). This same pattern was also observed with UgCl236 and UgCl422 infections in both A/J and C57BL/6J mice (Fig. S4). These findings indicate that neutrophils drive the innate immune response to hypervirulent infections in A/J mice.

**Fig 8:**
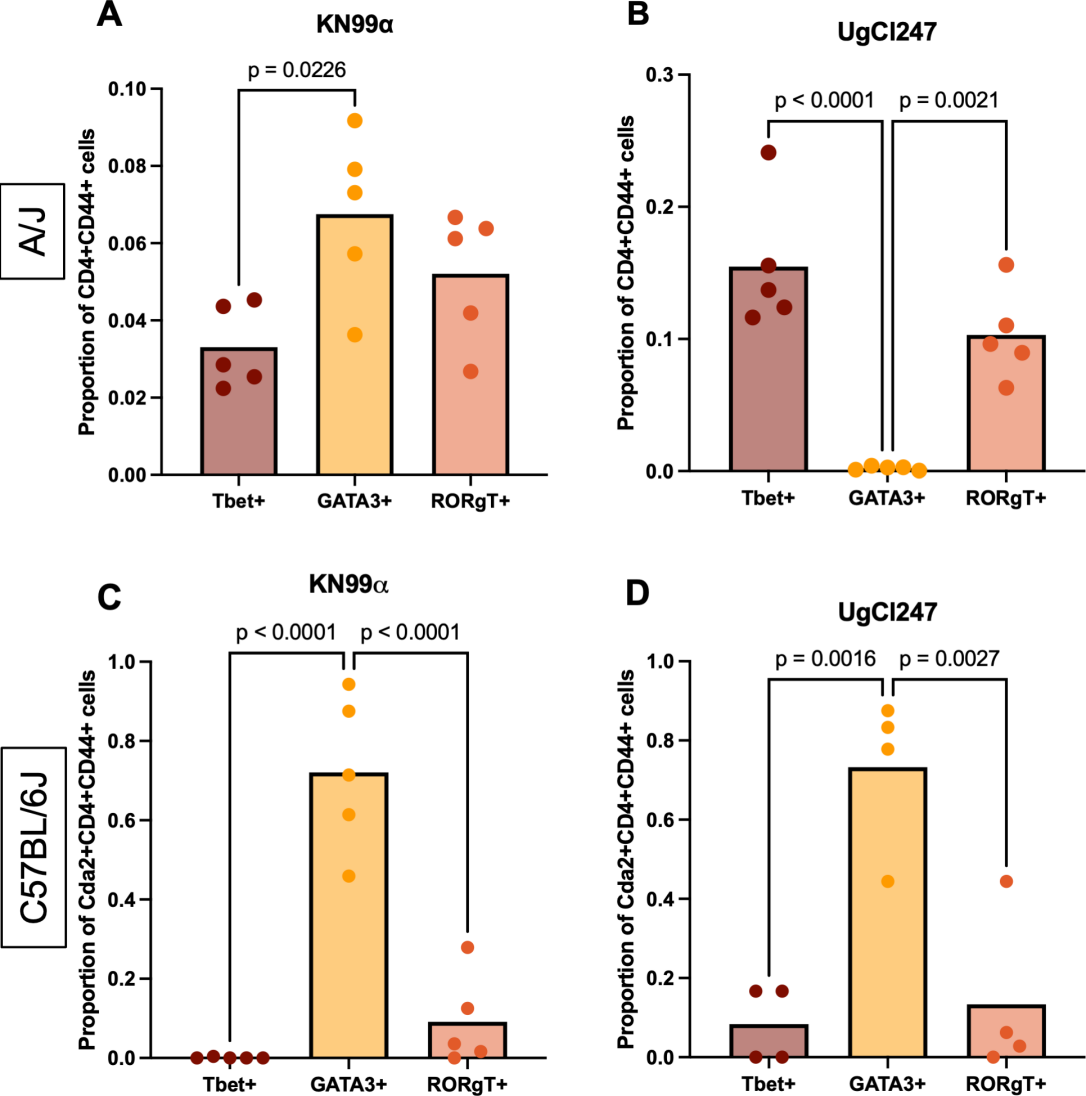
Differential CD4 T-cell polarization in response to UgCl247 infection in A/J and C57BL/6J mice. A/J mice were intranasally infected with *C. neoformans* (**A**) KN99ɑ (*n* = 5 mice) and (**B**) UgCl247 (*n* = 5 mice). C57BL/6J mice were intranasally infected with (**C**) KN99ɑ (*n* = 5 mice) and (**D**) UgCl247 (*n* = 4 mice). Single-cell lung suspensions were generated at 17 days post-infection. Proportions of CD4+ CD44+ FoxP3-Tbet+ (Th1), CD4+ CD44+ FoxP3-GATA3+ (Th2), and CD4+ CD44+ FoxP3-RORγ T+ (Th17) cells per lung were determined by flow cytometry. For C57BL/6J mice, a Cda2-MHCII tetramer was used to enrich for Cda2+-specific CD4+ CD44+ T cells. *P*-values were calculated by one-way ANOVA with Bonferroni correction.

Interestingly, increased T-cell responses to the hypervirulent isolates were observed in both A/J and C57BL/6J mice when compared with KN99α (Fig. 7A and B). Thus, we hypothesized that the adaptive immune response, specifically polarization of CD4 T-cells, may also be driving the hypervirulent phenotype in A/J mice. As expected based on previous studies (8), UgCl247 infection in C57BL/6J mice and KN99α infection in both A/J mice and C57BL/6J mice elicited significantly increased proportions of CD4+ CD44+ T cells expressing the Th2 transcription factor GATA3 (Fig. 8A, C and D). More importantly, we observed significantly increased proportions of activated CD4+ CD44+ T cells expressing the Th1 transcription factor Tbet and the Th17 transcription factor RORγT during UgCl247 infection (Fig. 8B). The same findings were also observed during UgCl236 and UgCl422 infections in A/J mice (Fig. S5). In addition, we used CD69+ and CD103+ to identify lung-resident CD4 T cells and measured CXCR3 expression, a chemokine receptor that is highly expressed on Th1 CD4 T cells (23). We found that the proportion of CD69+ CD103+ lung resident CD4+ T cells expressing CXCR3+was significantly higher during UgCl247, UgCl422, and UgCl236 infections compared with KN99α infection in A/J mice (Fig. S5). These results suggest that the pulmonary CD4 T-cell response during IFNγ-associated hypervirulent infection in A/J mice is due to Th1/17 polarization.

Altogether, these studies supported our theory that (i) the host factor(s) contributing to hypervirulence in the A/J mouse strain is due to unique genetic mutations/polymorphisms present only in A/J mice, and (ii) the Type 1/17 immune polarization observed in A/J mice promotes the hypervirulence phenotype.

### Variation in *C. neoformans* phenotypic responses to different inbred mouse strains

**Fig 9:**
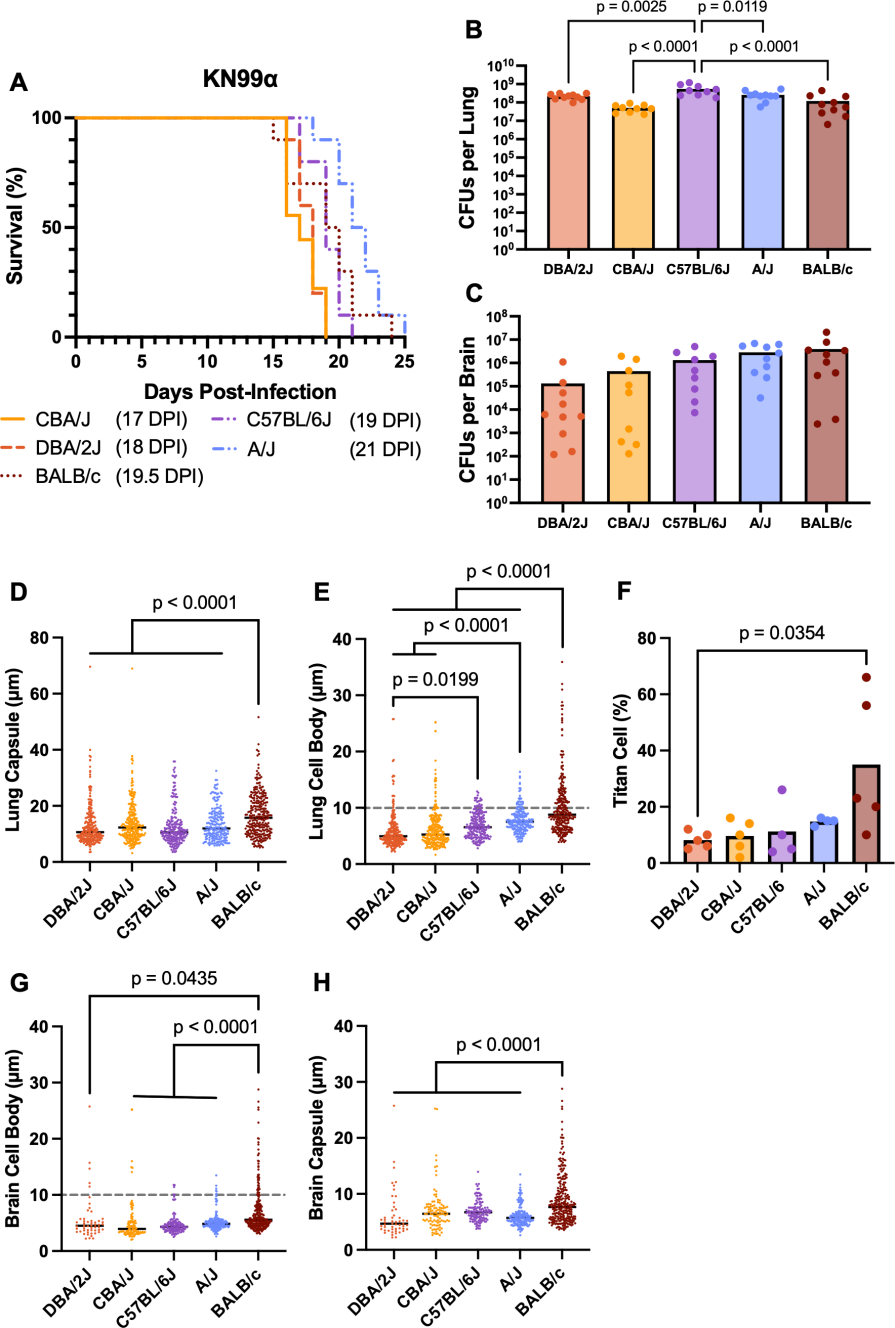
KN99α survival, cell body, and capsule size in A/J, C57BL/6, CBA/J, DBA/2J, and BALB/c mice. (**A**) A/J (*n* = 10), C57BL/6 (*n* = 9), CBA/J (*n* = 10), DBA/2J (*n* = 10), and BALB/c (*n* = 10) mice were intranasally infected with *C. neoformans* KN99ɑ. Mice were monitored for signs of morbidity. At the terminal endpoint, (**B**) the lungs and (**C**) brain of infected mice were harvested, homogenized, and plated for colony-forming units (CFUs). Kaplan–Meier survival kinetics were analyzed using log-rank testing, and survival curves were found to be significantly different with *P* < 0.0001. Median survival is denoted in parentheses by days post-infection (DPI). *P*-values calculated by one-way ANOVA with Bonferroni correction. Lungs and brain were also processed for cryptococcus cells, stained with India ink, and imaged. (**D**) Lung cell body diameter, (**E**) lung cell capsule size, (**F**) titan cell percentage, (**G**) brain cell body diameter, and (**H**) brain cell capsule size were determined for each cell per mouse. *P*-value was calculated by two-way ANOVA with Bonferroni correction.

During our interrogation of the hypervirulent isolates in various inbred mouse strains, we found it very intriguing that KN99α also had variable virulence in different mouse backgrounds that are considered immunocompetent. Indeed, the median survival of CBA/J, DBA/2J, C57BL/6J, BALB/c, and A/J mice infected with KN99α was significantly different from each other (Fig. 9A). While there was no difference in terminal brain fungal burden for the different mouse strains, C57BL/6J mice had significantly higher lung fungal burden compared with all the other mouse strains (Fig. 9B and C). We next measured the cryptococcal cell body size, capsule size, and titan cell percentage harvested from the lungs and brains of each mouse strain. Intriguingly, the inbred mouse strains that demonstrated significantly increased median survival (A/J and BALB/c) following KN99α infection also had correspondingly larger lung and brain cell body and capsule sizes (Fig. 9D through H). Conversely, inbred mouse strains with significantly decreased median survival (DBA/2J and CBA/J) with KN99α infection had smaller lung and brain cell body and capsule size (Fig. 9D through H). While both the capsule and cell body size were generally larger in A/J mice compared with C57BL/6J mice during infection with the genetically hypervirulent isolates, high variability in the phenotypes was observed across isolates (Fig. S6).

## DISCUSSION

Many peer-reviewed studies have linked cryptococcal meningitis patient outcomes to host- and pathogen-specific factors. From the host perspective, differential immune responses to cryptococcal meningitis have been identified as a major factor in patient outcome (2–5, 8, 24, 25). From the pathogen perspective, *C. neoformans* virulence (26), changes in cell size (27), and genotype (9–12, 28, 29) have all been found to have a profound impact on patient outcome. Yet, determining the relative contributions of the host and pathogen to *C. neoformans* disease progression and patient outcome continues to remain a challenge. The mouse inhalation model of *C. neoformans* infection is a useful tool for teasing apart the complexities of the infection by holding key pathogen and host factors constant. In this study, we identified three clinical isolates that cause hypervirulence in the inbred A/J mouse genetic background and studied the pulmonary immune response to these hypervirulent isolates in the A/J background (17). We then used a range of inbred mouse strains with different genetic backgrounds to understand how host-specific factors influence isolate virulence. Our findings lay important groundwork towards understanding the hierarchy of host- and pathogen-specific factors in determining disease outcome in *C. neoformans* infection.

In previous studies, we showed that infections in A/J mice recapitulate human disease variability and found that closely related *C. neoformans* clinical isolates displayed a wide range of virulence phenotypes when infected in A/J mice (15, 17). One such phenotype was hypervirulence, which we defined as median survival of 2 days before the median survival of the KN99α infection (17). In addition, the majority of the clinical isolates that were hypervirulent in A/J mice had significantly increased IFNγ production compared with KN99α infection (17). Mutations in the downstream region of the inositol transporter 4 (*ITR4*) gene leading to *ITR4* downregulation were associated with high IFNγ production and rapid mortality in A/J mice and may be a pathogen-specific factor responsible for hypervirulence in A/J mice (17). These findings suggested that *C. neoformans* cell wall, membrane, or capsule changes may be related to hypervirulence in A/J mice (17). However, aside from a pro-inflammatory/IFNγ signature, it was not clear what host-specific factors might be driving the observed hypervirulence.

In the current study, we showed that hypervirulence is a multi-faceted phenomenon that has both host and pathogen contributions. In addition, the pulmonary immune response to IFNγ-associated hypervirulent isolate infection in A/J mice was characterized by neutrophil infiltration and T-cell recruitment. Furthermore, we found that the hypervirulent isolate infections in A/J mice promoted a robust Type 1/17 proinflammatory response, as measured by increased CXCR3+ expression by lung tissue resident CD4+ T cells and increased Tbet+ and RORγT+ expression by activated CD4+ T cells. These findings were corroborated by increased IFNγ and IL-17A production in A/J mice.

The pro-inflammatory cytokines (IL-1β and TNF-α) that were increased during the hypervirulent infections suggest that the infected mice could be dying from a cytokine storm or immune reconstitution inflammatory syndrome (IRIS)-like pathology (30). This theory is especially compelling, as mice infected with the hypervirulent isolates have significantly decreased terminal lung and brain fungal burden, suggesting that the infected mice are not dying due to cryptococcal meningitis. The inbred A/J mouse strain also has notable genetic mutations related to immunity (21) that may make it more prone to developing IRIS or cytokine storm-like conditions. Notably, the hypervirulent isolates were obtained from patients with meningitis and advanced HIV who were greatly immunocompromised but survived the initial infection with *C. neoformans*. We could envision two scenarios in these patients: (i) the increased Th1/Th17 polarization in the context of immunosuppression generates a more protective immune response, or (ii) the enhanced Th1/Th17 polarization predisposes the patients to IRIS following immune reconstitution. A more extensive cohort of patients is needed to explore these scenarios in humans. Future studies that manipulate the A/J mouse immune response to mimic human disease may also shed light on the beneficial or detrimental impacts of these hypervirulent isolate infections.

We took advantage of inbred mouse strains with different immune and genetic backgrounds to identify host-specific factors that may contribute to hypervirulence in A/J mice. Using BALB/c, CBA/J, DBA/2J, and B6.Nlrc4-/- mice, we determined that the *Hc*^0^ mutation for complement deficiency, the *Nlrp1b^R/R^* allele, and the NAIP5/NLRC4 inflammasome pathway were not responsible for hypervirulence during infection. These data show that the characterized genetic mutations present in the A/J mouse background are not the cause of the hypervirulent phenotype. Instead, it is likely that the genetic differences between A/J and other inbred mouse strains that are influencing the hypervirulence phenotype have yet to be discovered.

During this study, however, we noted that A/J, C57BL/6J, BALB/c, CBA/J, and DBA/2J mice infected with KN99α had significantly different median survival times. These results suggest that the host inflammatory response against *C. neoformans* is also different between these mouse strains, as shown in other studies (31–33). We believe it is worth emphasizing that selection of appropriate inbred mouse strains should be a major consideration for future *C. neoformans* studies, especially as it pertains to the host inflammatory response (34).

When infected in C57BL/6J mice, the isolates were not hypervirulent when compared with KN99α and even promoted a Th2-dominant response with eosinophilic inflammation. Interestingly, a study looking at the difference between A/J and C57BL/6J mice in the host immune response to *Streptococcus suis* infection during septic shock-like syndrome found a similar differential immune response (35). Domínguez-Punaro et al. found that A/J mice were significantly more susceptible to *S. suis* infection compared with C57BL/6J mice, and A/J mice infected with *S. suis* had higher production of TNF-α, IL-12p40/p70, IL-1β, and IFNγ (35). Thus, the immunological difference we observed is replicated in other systems and is a major contributor to virulence in infectious models beyond just *C. neoformans*. It seems likely that uncharacterized genetic differences between A/J and C57BL/6J mice can influence their differential immune response against pathogens.

Overall, the mouse inhalation model can be used to identify both host- and pathogen-specific factors that affect *C. neoformans* disease progression. However, we must be careful when extrapolating the findings of this study to other hypervirulent isolates because clinical isolates are not genetically identical and can promote virulence via different pathways (14, 17). Regardless, the findings from this study lend additional credence to the damage response framework theory envisioned by Pirofski and Casadevall (36), where the host immune status is a key component that influences the clinical outcome of *C. neoformans* infection.

## MATERIALS AND METHODS

### Inbred mouse strains

The inbred mouse strains that were used in this study are C57BL/6J (Jackson Laboratory), A/J (Jackson Laboratory), BALB/c (Charles River Laboratories), DBA/2J (Jackson Laboratory), and CBA/J (Jackson Laboratory). B6.Nlrc4-/- was generated as previously described (37) and was kindly gifted by Russell E. Vance. All mice were housed in specific pathogen-free conditions. All mice used for the experiments in this study were 6–8 weeks of age; all controls were sex- and age-matched.

### Cryptococcus strains

The clinical isolates used in this study were collected in Uganda as part of the Cryptococcal Optimal ART Timing (COAT) trial (38) and subsequently analyzed in Gerstein et al. (14). The laboratory reference strain KN99α (39) was used as a control for the mouse experiments. Strains were maintained as glycerol stocks at −80°C and grown on yeast peptone dextrose (YPD) plates with 0.04 g/L chloramphenicol. The hypervirulent clinical isolates UgCl422, UgCl236, and UgCl247 used in this study were identified in previous studies (14, 17) and were defined as isolates that have a median survival of 2 days less than that of KN99α in A/J mice.

### Infection

*C. neoformans* strains were streaked on YPD + 0.04 g/L chloramphenicol agar plates for 48 h at 30°C prior to use. YPD broth was inoculated with colonies from the aforementioned plate and incubated for 16 h at 30°C and 225 RPM. The resulting inoculum was prepared by centrifuging the culture for 1 min at 14,000 RPM (17,968×*g*) to pellet the cells, washing the cells three times with phosphate-buffered saline (PBS; Gibco), and resuspending the cells in PBS at a concentration of 1 × 10^6^ cells/mL. A well-established intranasal pulmonary inhalation model of cryptococcosis was used for this study (39, 40). Then, 6–8-week-old, sex-matched mice were fully anesthetized with pentobarbital until mice did not respond to a toe pinch. Subsequently, 5 × 10^4^ *C. neoformans* cells in 50 µL of PBS (1 × 10^6^ cells/mL) were placed on the nares of each mouse, and the mice inhaled the inoculum into the lower respiratory tract. The mice were suspended by their incisors for 5 min and subsequently placed upright on a paper towel in their cage until regaining consciousness.

### Survival

Ten mice per experimental group were infected as described above. Animals were monitored for morbidity and euthanized via CO_2_ asphyxiation when endpoint criteria were reached. Endpoint criteria were defined at 20% total body weight loss, loss of 2 grams of weight in 2 days, or symptoms of neurological disease (i.e., domed head, seizures, partial paralysis, etc.).

### Organ fungal burden

Ten mice per experimental group were infected as described above. At the terminal endpoint, mice were euthanized. The lungs, brains, spleens, kidneys, livers, or hearts were isolated from mice via dissection and placed into 4 mL sterile PBS for the lungs and 2 mL sterile PBS for the brain. The collected tissues were homogenized, and serial dilutions of tissue homogenates were plated on YPD + 0.04 g/L chloramphenicol and incubated at 30°C. *C. neoformans* colonies were counted after 48 h of incubation.

### Histopathology

Mice were sacrificed at 17 days post-infection, as described previously. Whole lungs were collected, inflated, and fixed in 10% neutral-buffered formalin (Fisher Scientific, Waltham, MA). Fixed organs were paraffin-embedded, sectioned, slide mounted, and stained with hematoxylin and eosin (H&E) for standard histological visualization or with immunohistochemical antibodies for iNOS (1:175) and EPX (1:1000).

### *C. neoformans* cell body and capsule size measurements

Five mice per experimental group were infected as described above. At terminal endpoint, mice were euthanized. The lungs and brains were collected and homogenized at 4 or 2 mL PBS, respectively. The organs were transferred to another tube containing 1 mg/mL Collagenase Type 1 (Gibco) and incubated for 1 h at 37°C. Cells were washed three times in 0.05% SDS and filtered using a 70 µm filter. Following fixation in 3.7% formaldehyde and staining with India ink, cells were imaged using a ZEISS Axioskop microscope with differential interference contrast (DIC). For each mouse, at least 50 cells per lungs and 50 cells per brain were captured, when possible. The measurement of the diameter of each *Cryptococcus* cell body was obtained using ZEISS ZEN lite. Capsule size was generated by subtracting cell body diameter from the total cell diameter. Titan cells were defined as having a cell body larger than 10 µm. Titan cell percentage was determined by calculating the percentage of cells over 10 µm in diameter from each mouse.

### Cytokine analysis

Mice were infected as described above. At 17 days post-infection (DPI), 21 DPI, or terminal endpoint, mice were euthanized. Lungs were isolated via dissection and placed into 4 mL sterile PBS on ice. Lungs were homogenized, and 2 mL of homogenate was combined with protease inhibitor cocktail (Roche, Basal, Switzerland) on ice and centrifuged for 10 min at 10,000 RPM (9,188×*g*). The supernatant was transferred to a fresh tube and flash frozen with liquid nitrogen. The supernatant was stored at −80°C until cytokine analysis. Then, 20 µL of supernatant was added to the Th1/Th2/Th9/Th17/Th22/Treg Cytokine 17-Plex Mouse ProcartaPlex Panel (Thermo Fisher Scientific) and analyzed per the manufacturer’s instructions on a Luminex MAGPIX (Luminex Corporation).

### Flow cytometry analysis

For analysis of the pulmonary immune response, we utilized intravascular staining to discriminate between vascular and tissue leukocytes as described previously (41). Briefly, mice were intravenously injected via tail vein with BUV394-labeled CD45 antibody (30-F11, BD Biosciences) and were euthanized with CO_2_ 3 min following injection. Pulmonary leukocytes were isolated as described previously (42). Briefly, the chest cavity was opened, cold PBS was used to perfuse lungs via the right ventricle, and the trachea was exposed. The lungs were inflated with 2 mL of digestion solution containing 1.5 mg/mL Collagenase A (Roche), 5 mM DNase I (Ambion), 5% fetal bovine serum (FBS), and 10 mM HEPES (MP Biomedicals) in Hanks’ Balanced Salt Solution (HBSS, Gibco). The lungs were excised and placed in 5 mL digestion solution. Lung tissue plus digestion solution was incubated in a 37°C water bath for 30 min with gentle vortexing every 8–10 min. The resulting cell suspensions were strained through a 70 µM cell strainer and washed with 25 mL of PBS. Cells were then centrifuged for 10 min at 300×*g* for 10 min at 4°C.

For leukocyte isolation, the resulting cell pellet was resuspended in PBS containing 2% FBS and 1 mM EDTA, and CD45+ cells were isolated via positive selection using the EasySep Mouse CD45 Positive Selection Kit (StemCell) per manufacturer’s instructions.

For CD4 T-cell isolation, the cell pellet was resuspended in 40% Percoll-RPMI medium (GE Life Sciences). A Percoll density gradient was created (5 mL of 40% Percoll-RPMI top, 3 mL 67% Percoll-PBS bottom) and the samples were centrifuged for 20 min at 650×*g* without braking. The lymphocytes at the interface were removed, washed twice with PBS containing 1 mg/mL bovine serum albumin (BSA, Sigma) and 0.002 mM EDTA (Invitrogen), and centrifuged for 10 min at 300×*g*, 4°C. CD4 T cells were isolated via negative selection using the EasySep Mouse CD4+T cell Isolation Kit (StemCell).

Cda2-MHCII tetramers were generated (8) and produced (43) as previously described. Following lymphocyte isolation via Percoll gradient, 25 nM Cda2-MHCII-tetramer-PE was added to the sample and incubated at 25°C for 1 h in the dark. We omitted the negative CD4 T-cell isolation step (described above) in order to reduce the possibility of nonspecific binding during tetramer enrichment. Tetramer-bound cells were enriched using the EasySep PE Positive Selection Kit II (StemCell) following manufacturer’s instructions.

All single-cell suspensions were stained with Near-IR Live Dead viability dye (Biolegend) according to manufacturer’s instructions and then incubated for 15 min on ice with CD16/32 antibody (Biolegend) to prevent nonspecific antibody binding. For surface staining, samples were stained with fluorophore-labeled antibodies at 4°C for 30 min. If intracellular staining was not required, then samples were washed, fixed with equal parts IC fixation buffer (eBioscience) and cell staining buffer (Biolegend), and stored at 4°C until ready for data acquisition by flow cytometry. For intracellular staining, cells were washed, fixed, and permeabilized using the Foxp3/Transcription Factor Staining Buffer Set (eBioscience) according to the manufacturer’s instructions. Samples were stained intracellularly with fluorophore-labeled antibodies at 4°C overnight. After staining, cells were washed with 1× permeabilization buffer, placed in cell staining buffer, and stored at 4°C until ready for data acquisition by flow cytometry.

All data were acquired with a BD LSR Fortessa flow cytometer using BD FACSDiva software (BD Bioscience). Compensation was performed at the beginning of each experiment with UltraComp eBeads plus Compensation Beads (Invitrogen) and the ArC Amine Reactive Compensation Bead Kit (Life Technologies). Data were analyzed using FlowJo v10.8.1.

For pulmonary leukocyte response to *C. neoformans* infection, the following fluorophore-labeled antibodies were used: CD45 (30-F11, BUV805, Invitrogen), CD11b (M1/70, APC, BD Biosciences), CD11c (HL3, BV786, BD Biosciences), CD24 (M1/69, BV711, BD Biosciences), CD64 (X54-5/7.1, BV421, Biolegend), IA/IE (M5/114.15.2, BV605, BD Biosciences), Ly6C (HK1.4, PerCP-Cy5.5, eBioscience), Ly6G (1A8, AF700, BD Biosciences), Siglec F (E50-2440, PE-CF594, BD Biosciences), TCRβ (H57-597, PE, Biolegend), B220 (RA3-6B2, BV650, Biolegend), and NK1.1 (PK136, AF488, Biolegend). The gating strategy for DCs, macrophages, monocytes, eosinophils, T cells, B cells, and NK cells is shown in Fig. S7.

To calculate the number of immune cells per lung, the proportion of CD45+ leukocytes, monocytes, alveolar macrophages, interstitial macrophages, exudative macrophages, neutrophils, eosinophils, CD11b- DCs, CD11b + DCs, NK cells, B-cells, and T-cells was determined by flow cytometry. This percentage was then multiplied by the total number of lung leukocytes determined by hemocytometer.

For pulmonary T-cell response to *C. neoformans* infection, the following fluorophore-labeled antibodies were used: TCRγ/δ (GL3, PE, Biolegend), PD-1 (J43, APC, Invitrogen), NK1.1 (PK136, BV786, BD Biosciences), CD69 (H1.2F3, PerCP-Cy5.5, Invitrogen), CXCR3 (CXCR3-173, BV605, Biolegend), CD8 (53–6.7, BV711, Invitrogen), CD62L (MEL-14, BV421, Biolegend), CD44 (IM7, PE-CF594, BD Biosciences), TCRβ (H57-587, AF700, Biolegend), CD35 (PC61, BV650, Biolegend), CD103 (2E7, AF488, Biolegend), CD4 (GK1.5, BV805, BD Biosciences), B220 (RA3-6B2, APC-eFluor780, Thermo Fisher Scientific), CD11c (N418, APC-eFluor780, Thermo Fisher Scientific), CD11b (M1/70, APC-eFluor780, Thermo Fisher Scientific), and F4/80 (BM8, APC- eFluor780, Thermo Fisher Scientific). The gating strategy for CXCR3 expression on lung resident CD4 T cells is shown in Fig. S8.

To differentiate the CD4 T-cell subsets during *C. neoformans* infection, the following fluorophore-labeled antibodies were used: B220 (RA3- 6B2, APC-eFluor780, Thermo Fisher Scientific), CD11c (N418, APC-eFluor780, Thermo Fisher Scientific), CD11b (M1/70, APC-eFluor780, Thermo Fisher Scientific), F4/80 (BM8, APC- eFluor780, Thermo Fisher Scientific), NK1.1 (PK136, APC- eFluor780, eBioscience), CD3 (17A2, AF700, Biolegend), CD4 (GK1.5, BUV39, BD Biosciences), CD8 (53–6.7, BV650, Biolegend), CD44 (IM7, BV605, Biolegend), Tbet (4B10, BV421, Biolegend), GATA3 (16E10A23, AF647, Biolegend), RORγT (Q31-378, PE-CF594, BD Biosciences), and Foxp3 (FJK-16S, AF488, eBioscience). The gating strategy for Th1, Th2, Th17, Treg, and undefined cells is shown in Fig. S9. The CD4 T-cell subset proportions were calculated by dividing each CD4 subset by the total of Th1, Th2, Th17, and Treg cells.

### Statistics

Statistical analysis was performed with GraphPad Prism 9 software (La Jolla, CA). Kaplan–Meier survival curves were analyzed for statistical significance using low-rank testing. Power calculations were performed to assess appropriate sample size for all experiments. For cytokine analysis, an outlier analysis was used to identify and remove outliers. Data were analyzed using two-tailed *t*-test, one-way, or two-way ANOVA with Bonferroni adjustments for multiple comparisons when justified. *P*-values ≤ 0.05 were considered statistically significant. All data presented in this study are representative from a minimum of at least three independent experiments or biological replicates.

## SUPPLEMENTAL MATERIAL

Fig. S1 iai.00585-24-s0001.tiff Cytokine data of BALB/c, CBA/J, and DBA/2J mice.

Fig. S2 iai.00585-24-s0002.tiff Flow cytometry from A/J mice.

Fig. S3 iai.00585-24-s0003.tiff Flow cytometry from C57BL/6J mice.

Fig. S4 iai.00585-24-s0004.tiff Relative proportions of lung immune response.

Fig. S5 iai.00585-24-s0005.tiff CD4 polarization in A/J and C57BL/6J mice.

Fig. S6 iai.00585-24-s0006.tiff Cell body and capsule data.

Fig. S7 iai.00585-24-s0007.tiff Bulk leukocyte flow cytometry gating strategy.

Fig. S8 iai.00585-24-s0008.tiff Pulmonary resident CD4 T-cell cytometry gating strategy.

Fig. S9 iai.00585-24-s0009.tiff Th subsets flow cytometry gating strategy.

Supplemental material iai.00585-24-s0010.docx Supplemental figure legends.

ASM does not own the copyrights to Supplemental Material that may be linked to, or accessed through, an article. The authors have granted ASM a non-exclusive, world-wide license to publish the Supplemental Material files. Please contact the corresponding author directly for reuse.
