# Supplementary figures and images for: The hypervirulent Type-1/Type-17 phenotype of Cryptococcus neoformans clinical isolates is specific to A/J mice
Source: Infect Immun. 2025 Mar 3;93(4):e00585-24. doi: 10.1128/iai.00585-24 (PMC11977316; doi:10.1128/iai.00585-24)

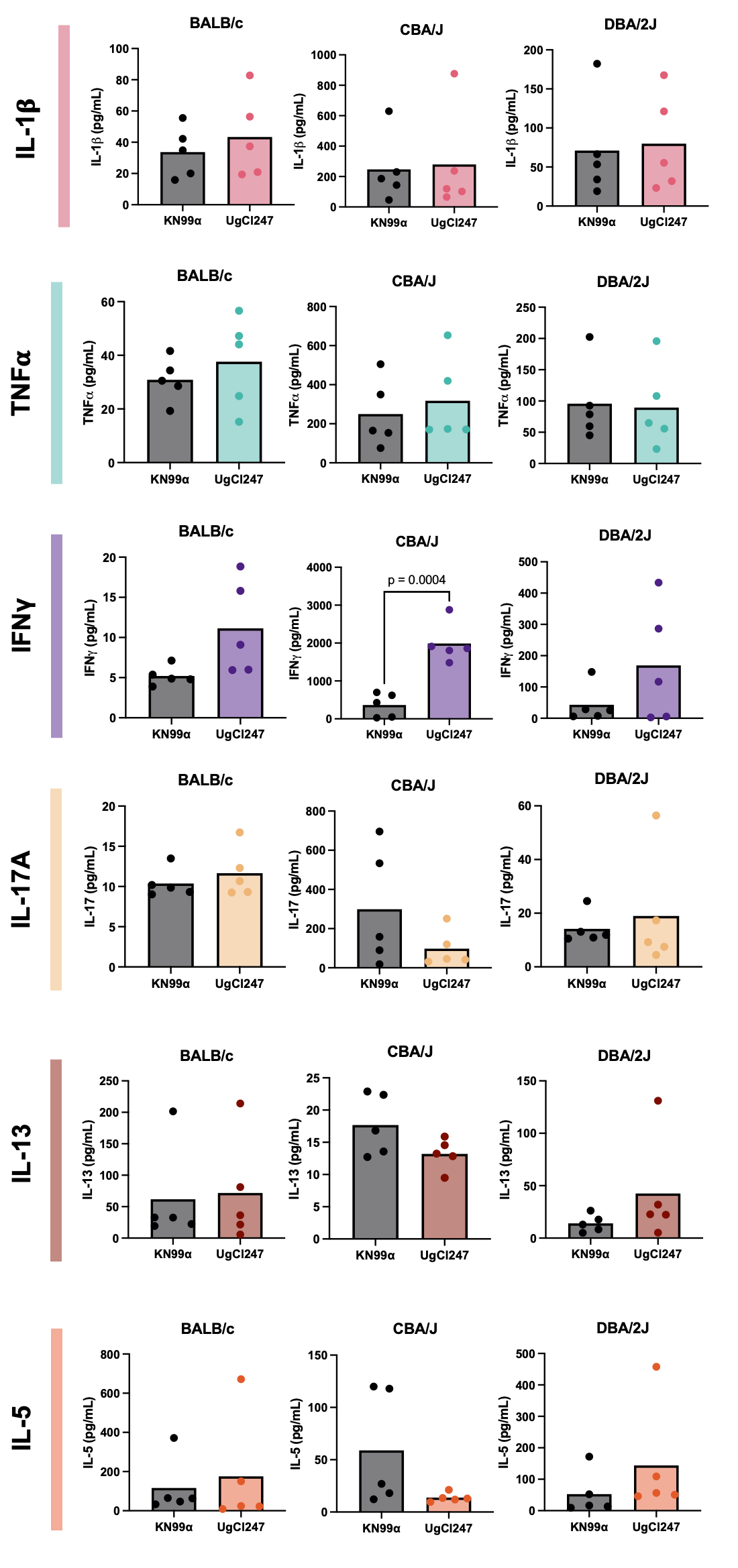

Supplement: Fig. S1 — Cytokine data of BALB/c, CBA/J, and DBA/2J mice. [file iai.00585-24-s0001.tiff]

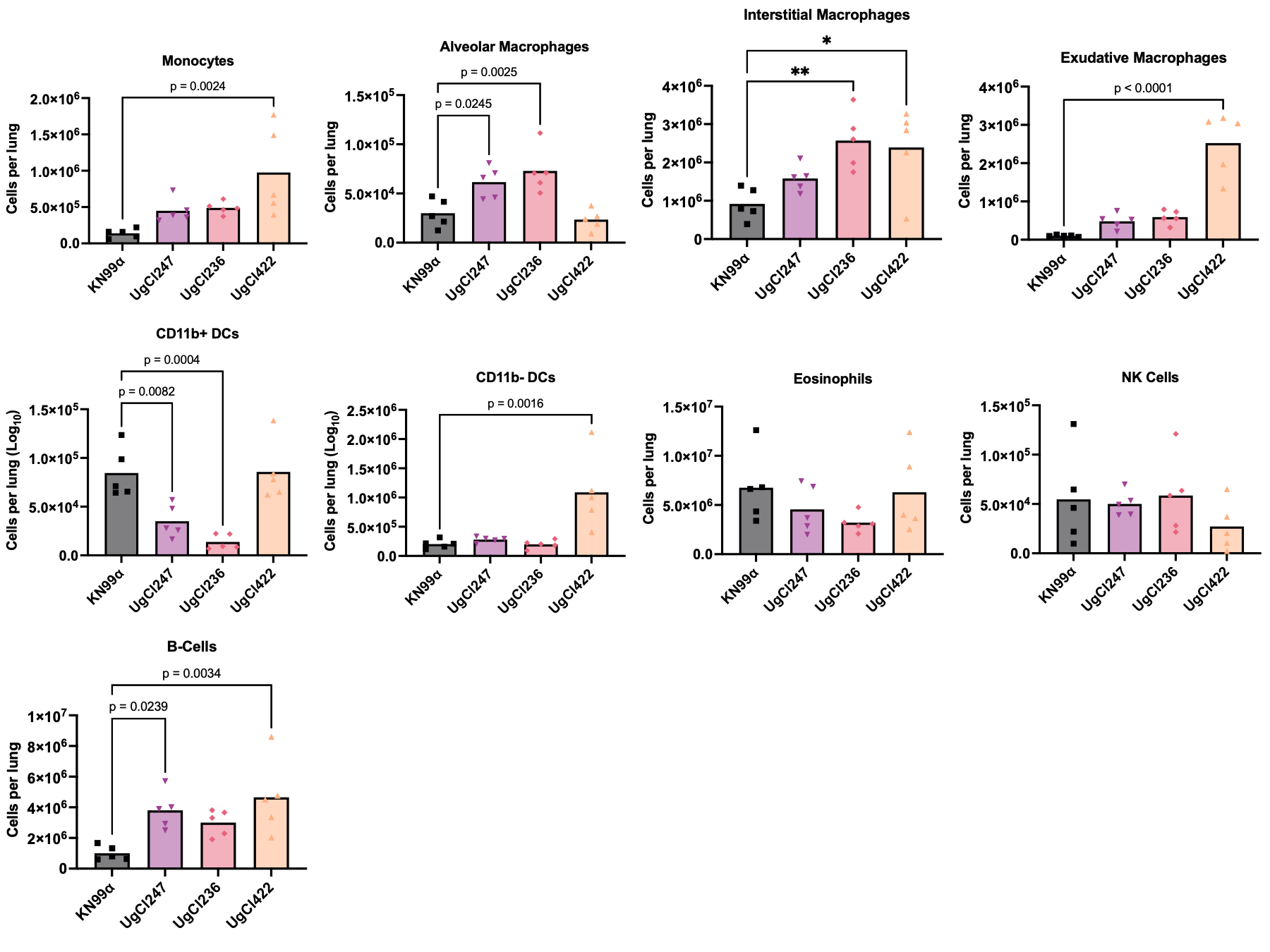

Supplement: Fig. S2 — Flow cytometry from A/J mice. [file iai.00585-24-s0002.tiff]

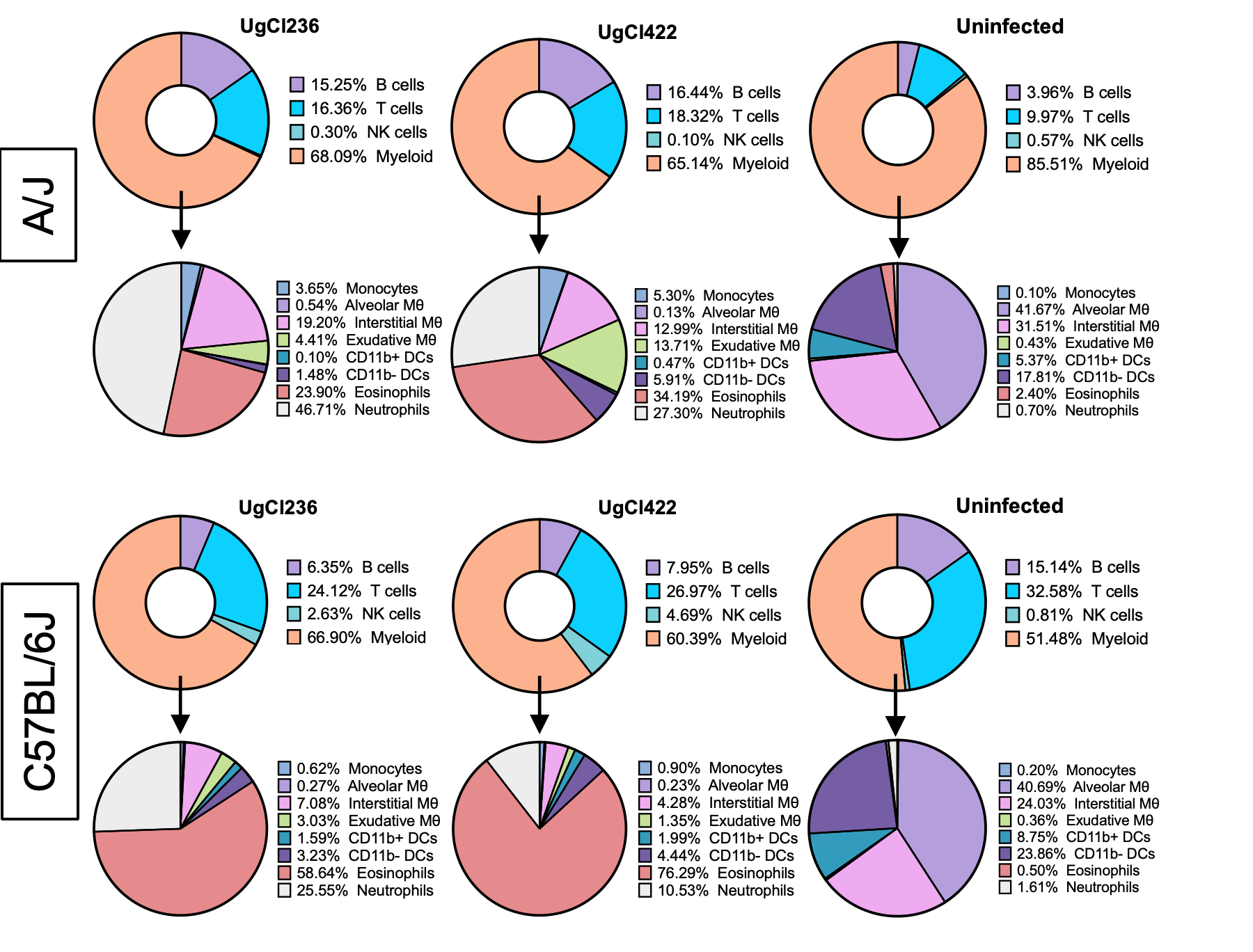

Supplement: Fig. S3 — Flow cytometry from C57BL/6J mice. [file iai.00585-24-s0003.tiff]

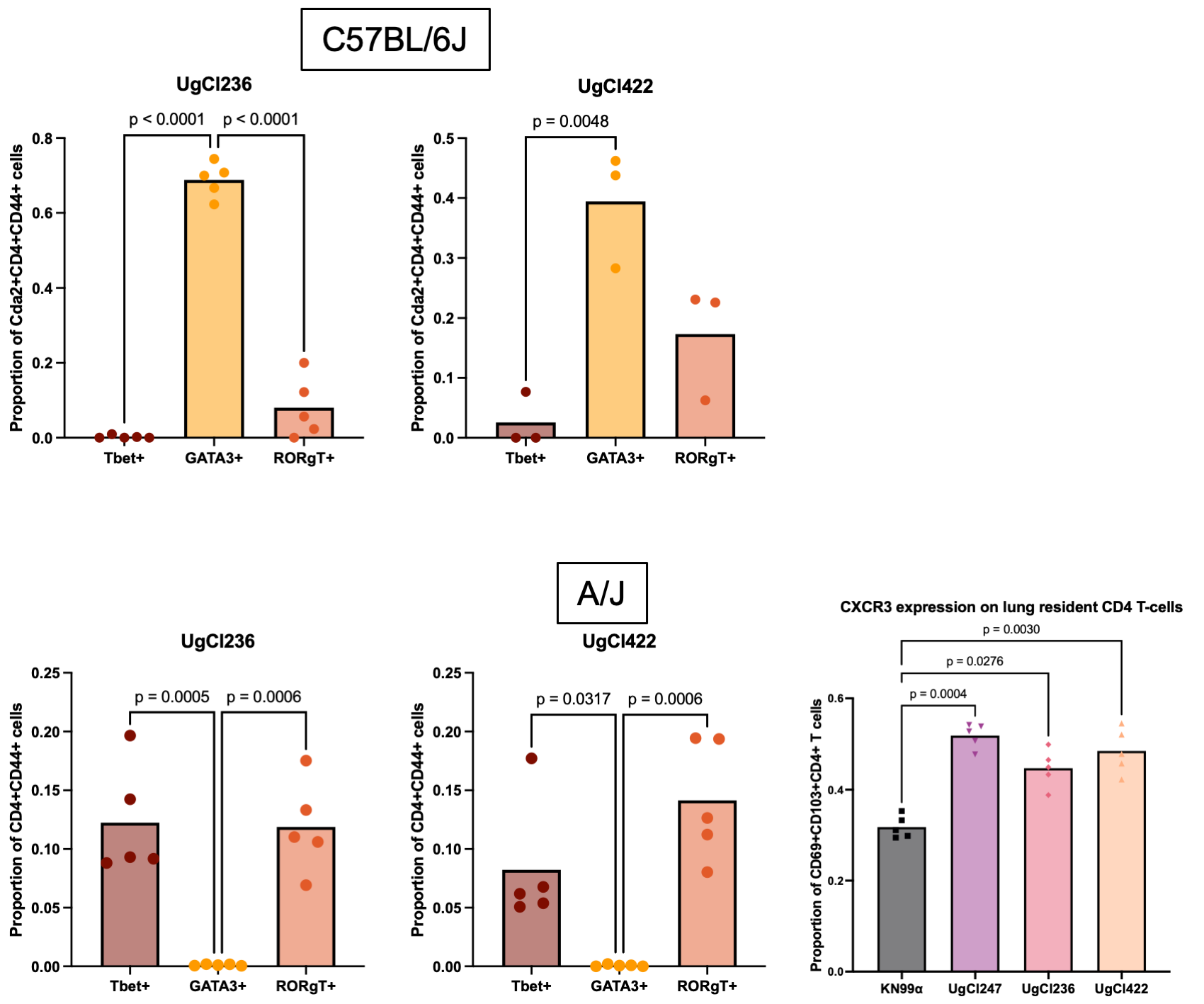

Supplement: Fig. S5 — CD4 polarization in A/J and C57BL/6J mice. [file iai.00585-24-s0005.tiff]

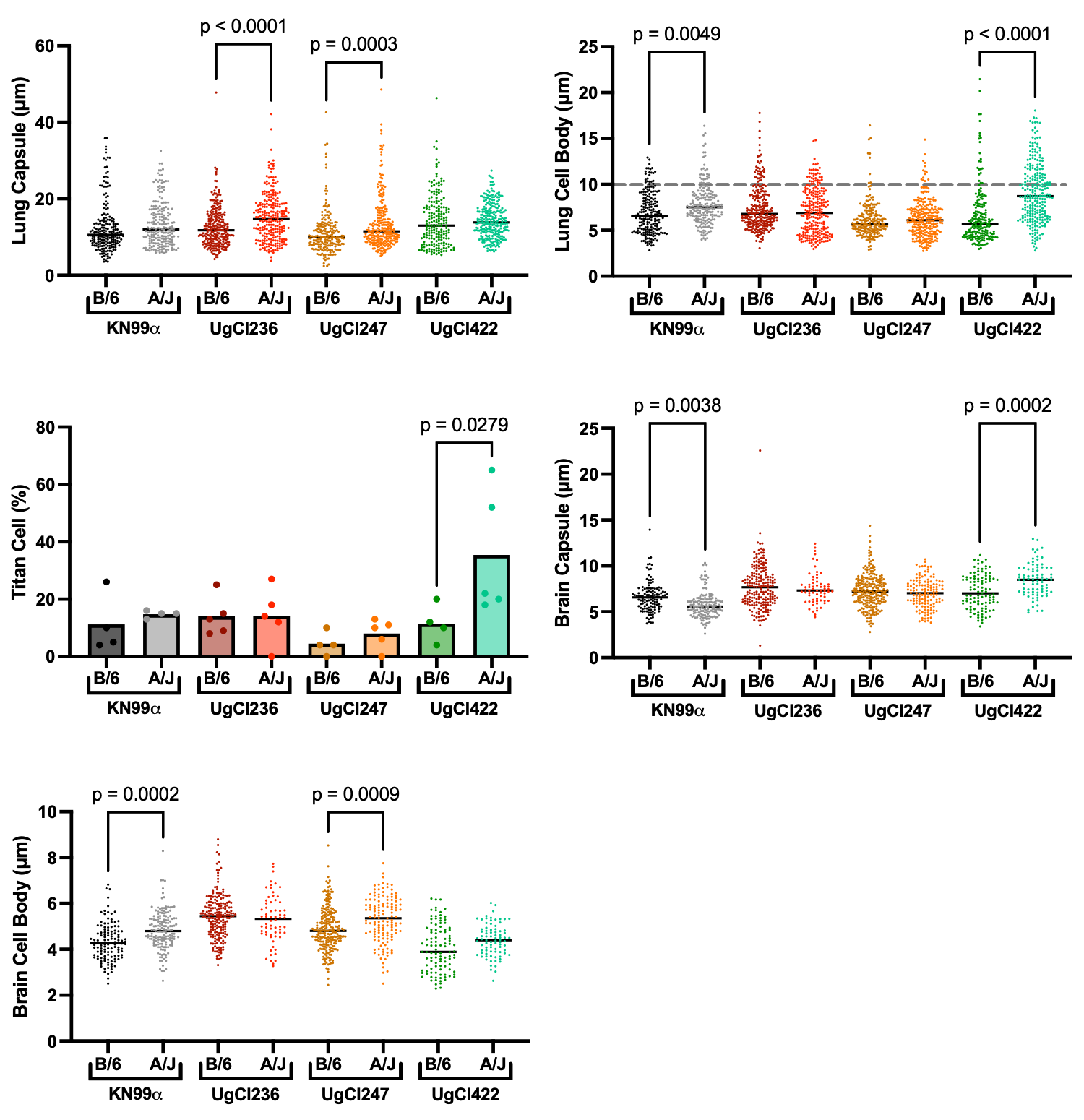

Supplement: Fig. S6 — Cell body and capsule data. [file iai.00585-24-s0006.tiff]

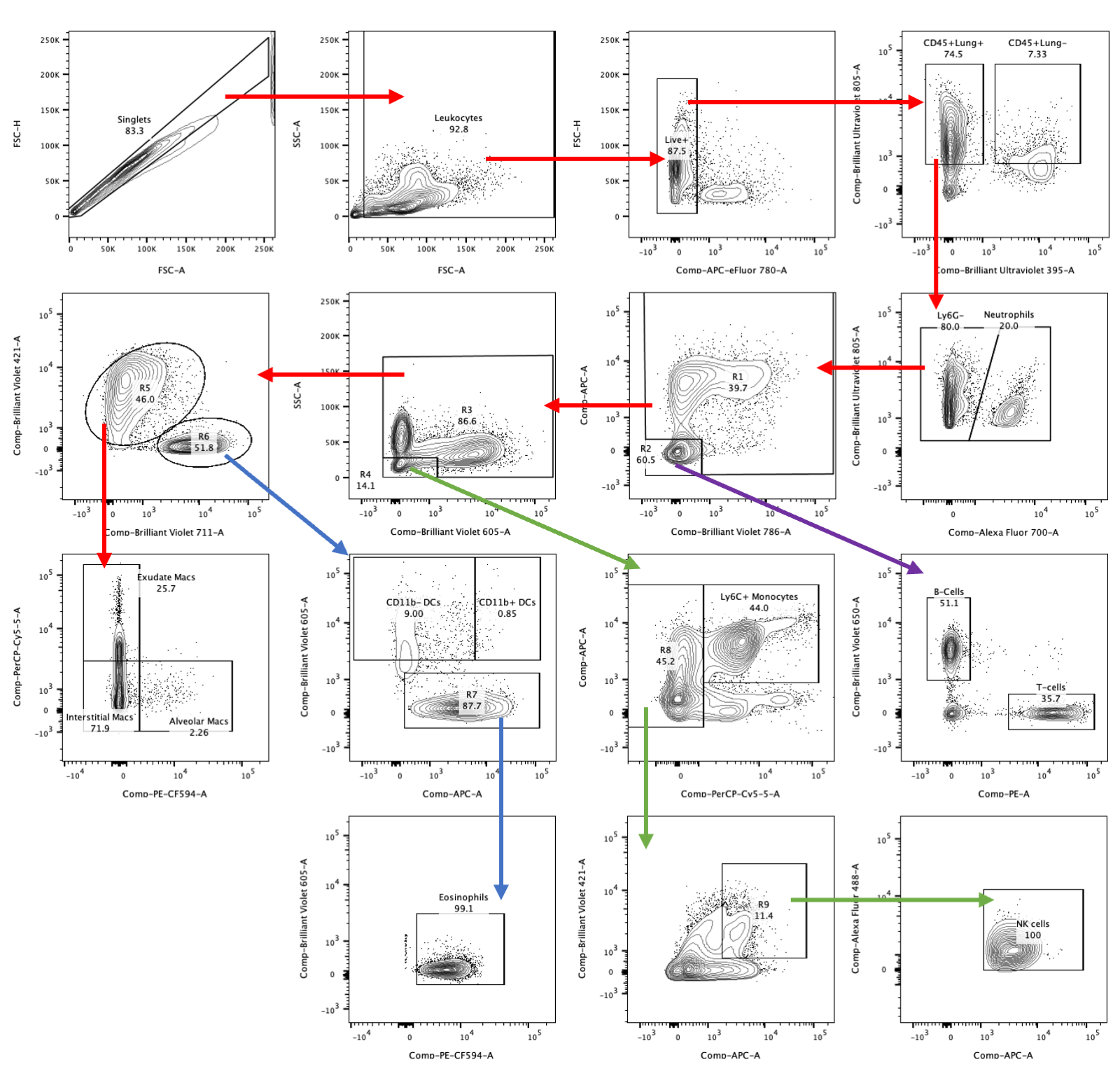

Supplement: Fig. S7 — Bulk leukocyte flow cytometry gating strategy. [file iai.00585-24-s0007.tiff]

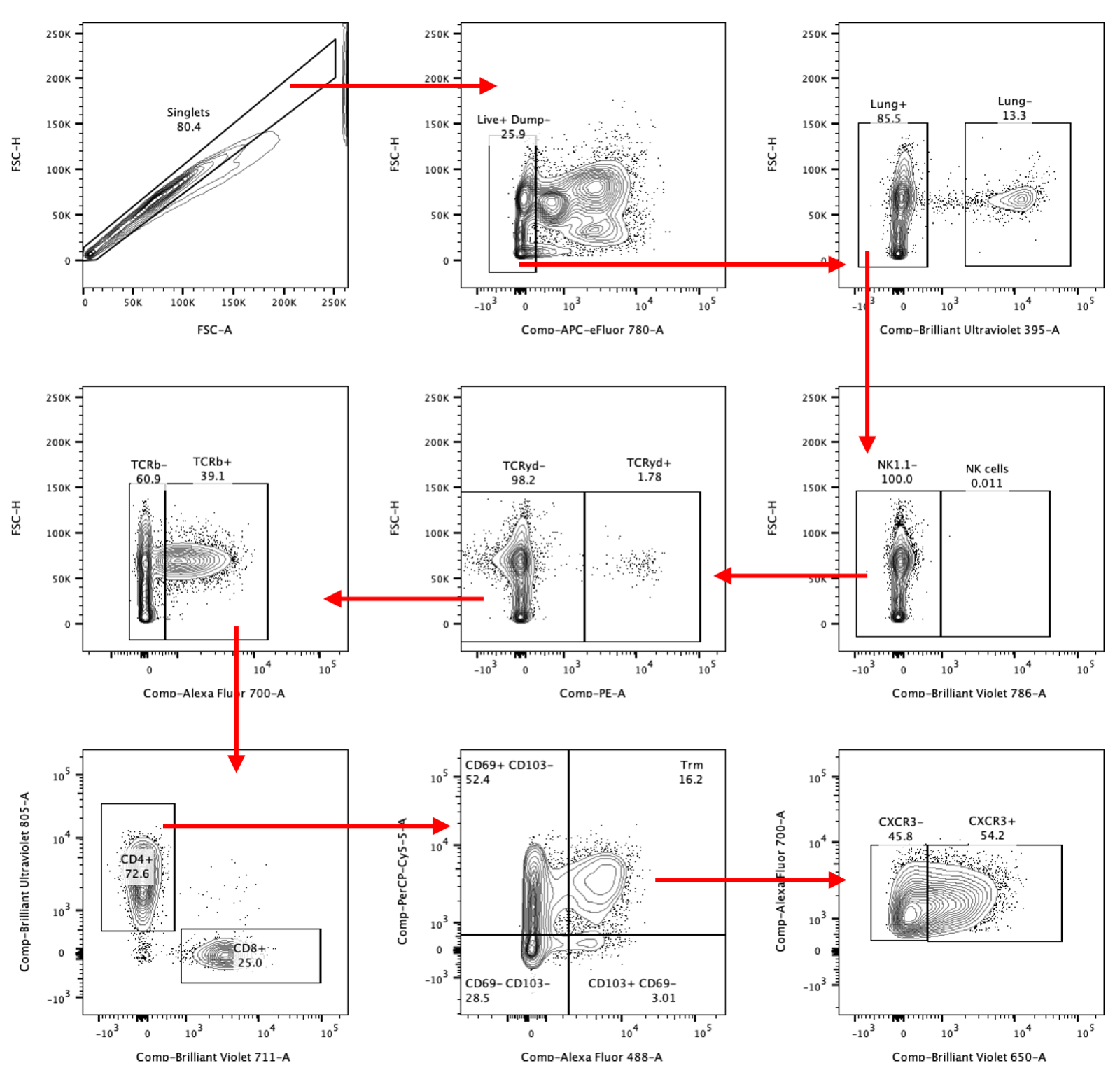

Supplement: Fig. S8 — Pulmonary resident CD4 T-cell cytometry gating strategy. [file iai.00585-24-s0008.tiff]

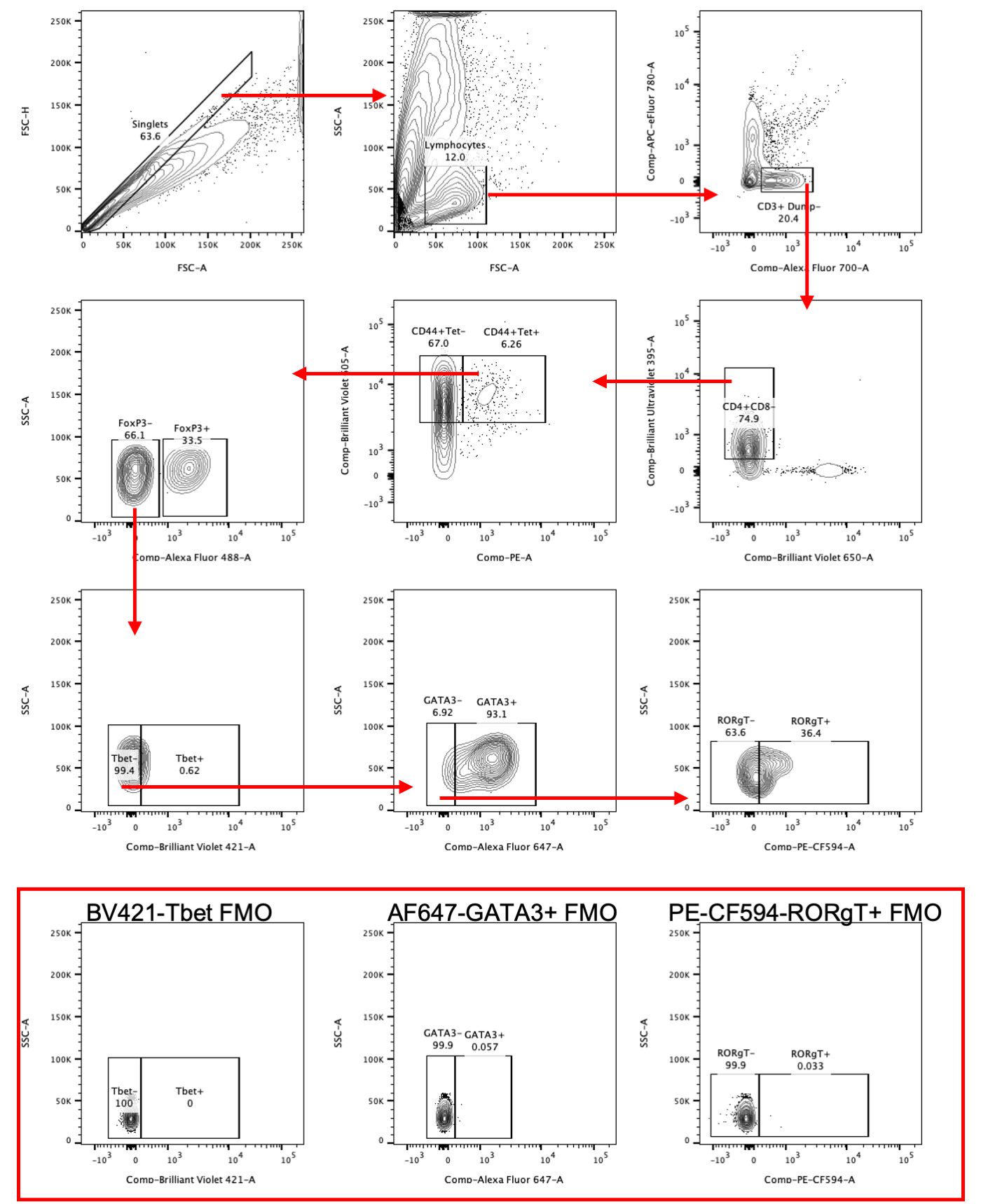

Supplement: Fig. S9 — Th subsets flow cytometry gating strategy. [file iai.00585-24-s0009.tiff]
