# Supplementary material for: The hypervirulent Type-1/Type-17 phenotype of Cryptococcus neoformans clinical isolates is specific to A/J mice
Source: Infect Immun. 2025 Mar 3;93(4):e00585-24. doi: 10.1128/iai.00585-24 (PMC11977316; doi:10.1128/iai.00585-24)
Supplement: Supplemental material — Supplemental figure legends. [file iai.00585-24-s0010.docx]

**Supplementary Figure 1: Cytokine response to UgCl247 infection in BALB/c, DBA/2J, and CBA/J mice.** BALB/c mice (n = 5 mice per strain), CBA/J mice (n = 5 mice per strain) and DBA/2J mice (n=5 mice per strain) were intranasally infected with *C. neoformans* UgCl247 or KN99ɑ. At 21 days post-infection or terminal endpoint (if prior to 21 DPI), mice were euthanized, lungs were isolated, homogenized, and lung supernatant was collected. Cytokine levels for IL-1𝛽, TNF-𝛼, IFNγ, IL-17A, IL-13, and IL-5 were measured using a Th1/Th2/Th9/Th17/Th22/Treg Luminex panel. P-value calculated by two-tailed t-test.

**Supplementary Figure 2: Pulmonary immune response to clinical isolates in A/J mice based on total cell count.** A/J mice were intranasally infected with *C. neoformans* UgCl247 (n = 5 mice), UgCl422 (n = 5 mice), UgCl236 (n = 5 mice), and KN99ɑ (n = 5 mice). Single cell lung suspensions were generated at 17 days post-infection. CD45+ leukocyte cell populations were quantified using flow cytometry. P-values calculated via one-way ANOVA with Bonferroni correction.

**Supplementary Figure 3: Pulmonary immune response to clinical isolates in C57BL/6J mice based on total cell count.** C57BL/6J mice were intranasally infected with *C. neoformans* UgCl247 (n = 4 mice), UgCl422 (n = 4 mice), UgCl236 (n = 4 mice), and KN99ɑ (n = 4 mice). Single cell lung suspensions were generated at 17 days post-infection. CD45+ leukocyte cell populations were quantified using flow cytometry. P-values calculated via one-way ANOVA with Bonferroni correction.

**Supplementary Figure 4: Pulmonary immune response to clinical isolates in A/J and C57BL/6J mice based on proportion.** Representative mean immune cell proportions of A/J infected with UgCl236 (n = 5 mice), UgCl422 (n= 5), and uninfected (n=4). Representative mean immune cell proportions of C57BL/6J mice infected with *C. neoformans* UgCl236 (n = 4 mice), UgCl422 (n= 4), and uninfected (n=3). Proportion of B-cells, T-cells, NK cells, and myeloid were calculated by dividing the total number of lung CD45+ cells. Proportion of monocytes, alveolar macrophages (M𝚹), interstitial M𝚹, exudative M𝚹, CD11b+ DCs, CD11b- DCs, eosinophils, and neutrophils were calculated by dividing the total number of myeloid cells.

**Supplementary Figure 5: UgCl236 and UgCl422 infection in A/J mice promoted Th1/Th17 polarization.** C57BL/6J and A/J mice were intranasally infected with *C. neoformans* UgCl236 (n = 5 mice) and UgCl422 (n = 3-5 mice). Single cell lung suspensions were generated at 17 days post-infection. Flow cytometric analysis showing total number of CD4+CD44+FoxP3-Tbet+ (Th1), CD4+CD44+FoxP3-GATA3+ (Th2), and CD4+CD44+FoxP3-ROR𝛾T+ (Th17) cells per lung for UgCl236 and UgCl422. Flow cytometric analysis showing the proportion of CXCR3+ cells out of total CD69+CD103+CD4+ T-cells (lung resident CD4 T-cells) during KN99ɑ, UgCl247, UgCl236, and UgCl422 infection in A/J mice. P-values calculated by one-way ANOVA with Bonferroni correction.

**Supplementary Figure 6: Mouse background influences cell and capsule size of hypervirulent isolates in mouse lungs and brain.** C57BL/6J (B/6) and A/J mice were intranasally infected with *C. neoformans* KN99ɑ, UgCl236, UgCl247, and UgCl422. At terminal endpoint, lungs and brain were collected, processed for cryptococcus cells, stained with India ink, and imaged. Lung cell body diameter, lung cell capsule size, titan cell percentage, brain cell body diameter, brain cell capsule size, and was determined for each cell per mouse. P-value was calculated by two-way ANOVA with Bonferroni correction.

**Supplementary Figure 7: Bulk leukocyte flow cytometry gating strategy.** Representative flow scatter plots of single cell suspension isolated from lungs of an A/J mouse at 17 days post-infection with *C. neoformans* UgCl247

**Supplementary Figure 8: Pulmonary resident CD4 T-cell cytometry gating strategy.** Representative flow scatter plots of single cell suspension isolated from lungs of an A/J mouse at 17 days post-infection with *C. neoformans* UgCl247

**Supplementary Figure 9: Th subsets flow cytometry gating strategy.** Representative flow scatter plots of single cell suspension isolated from lungs of an C57BL/6 mouse at 17 days post-infection with *C. neoformans* KN99𝛼. Fluorescence minus one (FMO) controls were used to determine gating for BV421-Tbet, AF647-GATA3, and PE-CF594-RORgT populations. C57BL/6 mice infections had additional MHCII-Cda2 tetramer+ enrichment step that was not performed in A/J mice infections.
